# Supplementary material for: Ionic liquid facilitated melting of the metal-organic framework ZIF-8
Source: Nat Commun. 2021 Sep 29;12:5703. doi: 10.1038/s41467-021-25970-0 (PMC8481281; doi:10.1038/s41467-021-25970-0)
Supplement: Supplementary file 1 — Supplementary Information [file 41467_2021_25970_MOESM1_ESM.pdf]

Supplementary information for

Ionic Liquid Facilitated Melting of the Metal-Organic Framework ZIF-8

Vahid Nozari[1], Courtney Calahoo[1], Joshua M. Tuffnell[2], David A. Keen[3], Thomas D. Bennett[2] and Lothar Wondraczek[1,4*]

[1]Otto Schott Institute of Materials Research, University of Jena, Jena, Germany

[2]Department of Materials Science and Metallurgy, University of Cambridge, Cambridge, United Kingdom

[3]ISIS Facility, Rutherford Appleton Laboratory, Harwell Campus, Didcot, Oxfordshire, United Kingdom

[4]Center of Energy and Environmental Chemistry, University of Jena, Jena, Germany

*Corresponding author, e-mail: lothar.wondraczek@uni-jena.de

Supplementary methods.

This file contains Supplementary discussion of NMR results, Supplementary discussion of washing experiments, fiber-coupled optical absorption studies, and Supplementary Figures 1 to 36, Supplementary Tables 1 to 9 and Supplementary References 1-12.

^{1}H NMR results of ZIF-8 protons.

Results for ZIF-8 protons are presented in Supplementary Figure 10 and Supplementary Tables S4-S6. The $H_{4,5}/H_1$ ratio in Supplementary Table S5 shows that after IL incorporation the ratio for pristine ZIF-8 (0.67 ± 5%) is preserved in IL@ZIF-8 sample (0.66 ± 5%) showing that the ZIF-8 linker is intact in the IL@ZIF-8 composite. However, we observed a decrease in this ratio to 0.59 ± 5% and 0.55 ± 5% in a_g(IL@ZIF-8-LT) and a_g(IL@ZIF-8-HT) glasses, respectively. Supplementary Table S6 illustrates the ratio of H_1 and $H_{4,5}$ protons in the IL@ZIF-8 composite and the glasses, with respect to the pristine ZIF-8. The percentage of $H_{4,5}$ protons are decreased from 65.29 % of IL@ZIF-8 to 59.09 and 48.10 % for a_g(IL@ZIF-8-LT) and a_g(IL@ZIF-8-HT), respectively. However, the percentage decrease in H_1 protons is less; almost no change was detected for a_g(IL@ZIF-8-LT) (66.79 %) and it decreased to 58.15 % for a_g(IL@ZIF-8-HT).

^{1}H NMR results of IL protons.

The NMR results of IL protons shows complex behavior for a_g(IL@ZIF-8-LT) and a_g(IL@ZIF-8-HT), as can be seen from Supplementary Figures 11a, 12a, 13a, 14a, and 15a. Overall, there are major changes in the chemical shifts of the IL protons (see Supplementary Table 7); the appearance of additional sets of peaks proves significant changes in electronic environment and the occurrence of new environments upon heating. The predicted ^{1}H NMR shifts of the protons in EMIM, considering all the possible decompositions/detachments of alkyl groups in Supplementary Figures 11b, 12b, 13b, 14b, and 15b, suggest that the appearance of the new peaks is consistent with partial decomposition of the IL. Supplementary Table S8 indicates intensity of each EMIM proton in the samples and compares the intensities with those observed for IL@ZIF-8.

The results show that the EMIM-h and EMIM-a environments in EMIM, are significantly changed within the a_g(IL@ZIF-8-LT) and a_g(IL@ZIF-8-HT) samples, whereas those in EMIM-b & c remain

mostly intact. This is in accordance with literature results, showing that the cation and anion interaction in imidazolium-based ILs occurs via the EMIM-h position. EMIM-h is the most acidic hydrogen in the cation of imidazolium-based ILs, responsible for directional hydrogen bonds between the ions. It can be used as a probe for determining the strength of interionic interactions.[1,2] The lower amount of EMIM protons in a_g(IL@ZIF-8-HT) compared to a_g(IL@ZIF-8-LT) indicates a greater degree of decomposition of the IL molecules in a_g(IL@ZIF-8-HT), consistent with TG-MS results.

^{13}C NMR single-pulse results.

Supplementary Figure 16 displays the single-pulse quantitative ^{13}C NMR spectra of the four samples: ZIF-8, IL@ZIF-8, a_g(IL@ZIF-8-LT) and a_g(IL@ZIF-8-HT), while Supplementary Figures 17 – 19 show the ppm ranges which correspond to the three main carbon peaks from the mIm linker of ZIF-8: CH_3 (C_1), CH ($C_{4,5}$) and C (C_2), respectively.[3] The IL peaks are discernible as extremely sharp doublets or triplets (*see* Supplementary Figure 20 for assignments[4]), while the ZIF-8 peaks tend to be broader. In the single-pulse ^{13}C NMR of the a_g(IL@ZIF-8-LT), Supplementary Figure 16, it is very clear that many of the ZIF-8 peaks have broadened (with some sharper peaks indicative of crystallinity at LT), while any evidence of the sharp IL peaks is missing, indicating the loss or immobilization of the IL carbons. Finally, in Supplementary Figure 17, only broad peaks exist at the HT condition for the a_g(IL@ZIF-8-HT) sample.

Although cross-polarization (CP) is inherently non-quantitative and sensitive to the proximity and amount of protons, single-pulse ^{13}C NMR can provide information on the amount of IL that has been incorporated into ZIF-8. The deconvolution of both methyl groups of the IL cation and ZIF-8 (C_e of EMIM and C_1 of mIm linker) in Supplementary Figure 20 shows 16 mol% IL and 84 mol% ZIF-8, respectively, which corresponds to about 26 wt% loading of IL. Although this is lower than expected from the synthesis procedure, 26 wt% is still a high loading. For quantification of the ZIF-8 carbons, it is important that the magnetization relaxes entirely; anything less than complete relaxation will likely result in underestimation of the ZIF-8 content. ^{13}C NMR in solids, especially when not attached to any protons, can have surprisingly long relaxation times, $T_1 > 20$ s. It is clear that there are some discrepancies for the %loading depending on the chosen analysis technique: TGA, ^{1}H NMR and ^{13}C NMR find 34.3 wt%, 21 wt%, and 26 wt% loading, respectively.

Unfortunately, all of these techniques have challenges making them less-than-ideally suited for accurate determination of wt% loading in these types of systems. TGA analysis does not differentiate between whether the IL in on the surface or in the pores, while quantitative ^{13}C NMR is challenging due to long relaxation times. Solution ^{1}H NMR using D_2O/DCl can result in the exchange of any acidic proton with deuterium, rendering that proton signal invisible to NMR.[5] For example, Hasani *et al.*[6] used a $H_2O + D_2O$ mixture to observe the exchange rate between water and the acidic NH proton on ethylimidazolium. In summary, a reliable measure of loading inside of the pores for IL@MOF composites still remains to be found.

Washing experiments on a_g(IL@ZIF-8-LT) and a_g(IL@ZIF-8-HT).

We investigated the removal of unreacted/decomposed IL from the a_g(IL@ZIF-8-HT) and a_g(IL@ZIF-8-LT) samples by washing the samples using acetone as the solvent. Details on the washing procedure are provided in the Methods section. In the IR spectra of the filtrates obtained from washing a_g(IL@ZIF-8-LT) and a_g(IL@ZIF-8-HT) samples, *see* Supplementary Figure 29, new bands were detected in the highlighted regions of the filtrates as compared to clean acetone, indicating partial uptake of soluble IL-related compounds from the a_g(IL@ZIF-8-LT) and a_g(IL@ZIF-8-HT) samples. Band positions of the newly emerged peaks in the filtrate are in the same regions where peak intensities/positions are different in the washed spectra. Microscope images of the washed a_g(IL@ZIF-8-LT) and a_g(IL@ZIF-8-HT) are presented in Supplementary Figure 30. They reveal smooth glassy surfaces with sharp edges, indicating that the samples remained stable during the washing experiment. Removal of some part of the decomposed IL is also evident from the color of the washed samples, which become notably more clear as compared to their appearance before washing (Figure 3 of the main text). Correspondingly, the filtrates (Supplementary Figure 30c) exhibit a yellow-brownish tint, which we take as further evidence for the washing-off of at least a part of the decomposed species from a_g(IL@ZIF-8-LT) and a_g(IL@ZIF-8-HT) samples. The glassy nature of washed a_g(IL@ZIF-8-HT) was examined by performing DSC-TGA cyclic experiments as shown in Supplementary Figure 31. A clear glass transition was observed on both upscans, however, due to extended exposure of the glass to high temperature at the first upscan, a shift of T_g to higher temperature (~ +5 °C) occurred on the second upscan.

Furthermore, we conducted N_2 and CO_2 adsorption experiments on unwashed and washed a_g(IL@ZIF-8-HT) and a_g(IL@ZIF-8-LT) samples (washed samples are denoted as post-washing "PW"). The N_2 and CO_2 adsorption isotherms presented in Supplementary Figure 32 show a notably enhanced gas uptake after washing. The increase in total pore volume is about fourfold in both the N_2 and the CO_2 experiment as compared to unwashed a_g(IL@ZIF-8-HT) and a_g(IL@ZIF-8-LT). In terms of N_2 uptake at 77 K, the washed samples clearly outperform other MOF glasses such as a_gZIF-62 and a_gZIF-76-mbIm, where no uptake was observed at 77 K in those glasses.[7,8] For CO_2 uptake at 273 K and 1 bar, a_g(IL@ZIF-8-HT) and a_g(IL@ZIF-8-LT) show 10 and 12 cc (STP) g^{-1} which is lower than 18 cc (STP) g^{-1} reported for a_gZIF-62.[9] However, the washed a_g(IL@ZIF-8-HT)-PW and a_g(IL@ZIF-8-LT)-PW samples (with 24 and 29 cc (STP) g^{-1}, respectively) again outperform data reported for a_gZIF-62 and a_g[(ZIF-8)$_{0.2}$(ZIF-62)$_{0.8}$] (18.7 cc (STP) g^{-1}) at similar temperature and pressure.[7]

Optical absorbance of a_g(IL@ZIF-8-HT) glass.

Optical properties of the a_g(IL@ZIF-8-HT) glass were analyzed by measuring the absorbance spectrum of an a_g(IL@ZIF-8-HT) glass film on a platinum surface, shown in Supplementary Figure 35a, using a fiber-coupled spectrometer. We selected an area where the thickness is sufficient so that interference effects can be neglected. In Supplementary Figure 35a, interference colors on the edges of the sample can be seen due to multiple reflections between the platinum surface and the glass substrate. Laser scanning microscopy determined a sample thickness of around 6 μm at the point of measurement. The topography of the glass film and the thickness profile can be seen in Supplementary Figures 35b and 35c, respectively. Assuming that the reflectivity of platinum is approximately 1 and that the reflection and transmission losses on the glass surface are negligible, we obtain the absorbance $(\lambda) = \log [R(\lambda)] / (2d)$, where $R(\lambda) = \phi_{T(\lambda)} / \phi_{P(\lambda)}$ (*see* Supplementary Figure 35d), which is the ratio between the flux reflected from an area covered with the glass and the flux reflected from an uncovered platinum surface. The measurement of the primary reflection could be suppressed by focusing through the glass on the glass-platinum interface. The light source was a deuterium arc lamp, covering a wide spectral range as shown in Supplementary Figure 35e. This allowed measuring reflection spectra from the sample in the range from 360 nm to 700 nm, as illustrated in Supplementary Figure 35f.

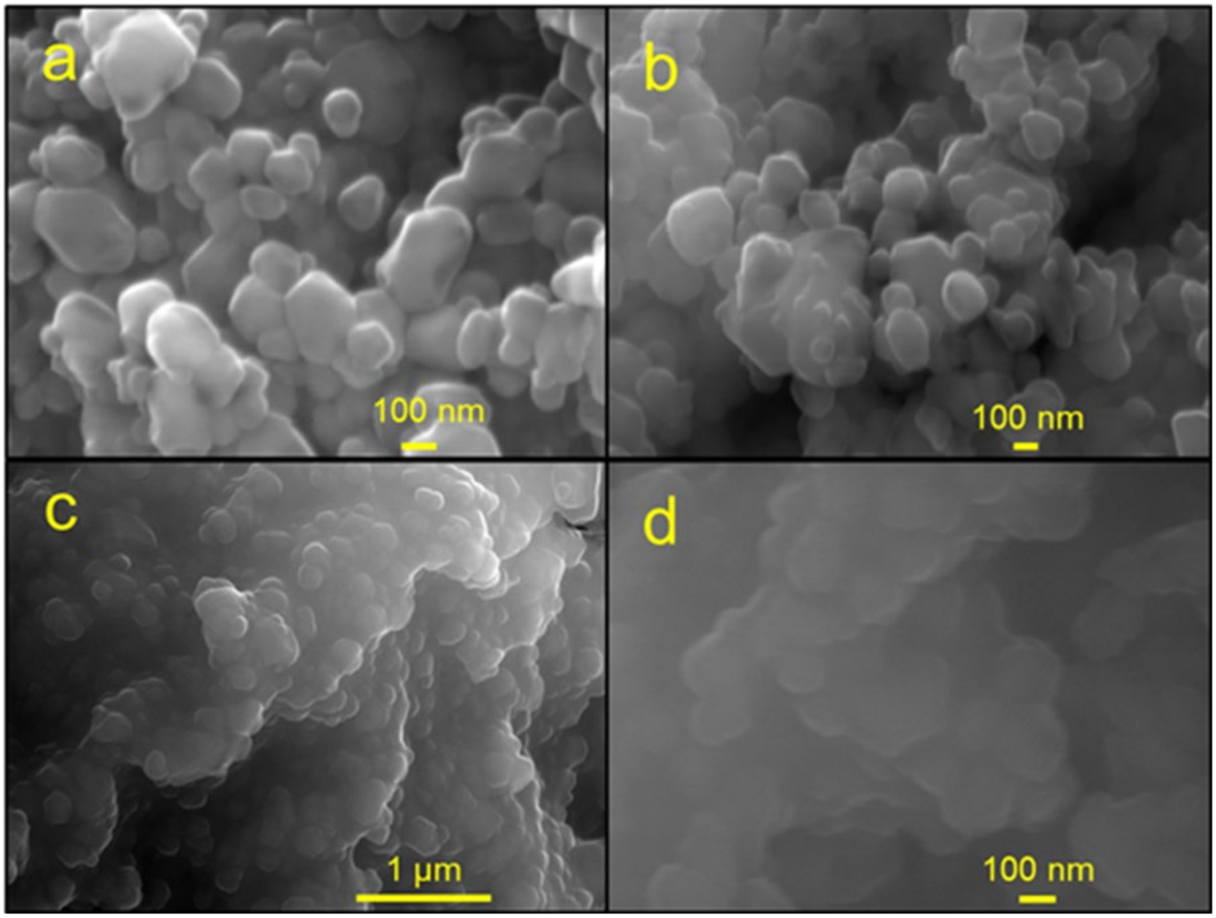

Supplementary Figure 1. SEM images of ZIF-8 and IL@ZIF-8. (**a**, **b**) ZIF-8. (**c**, **d**) IL@ZIF-8.

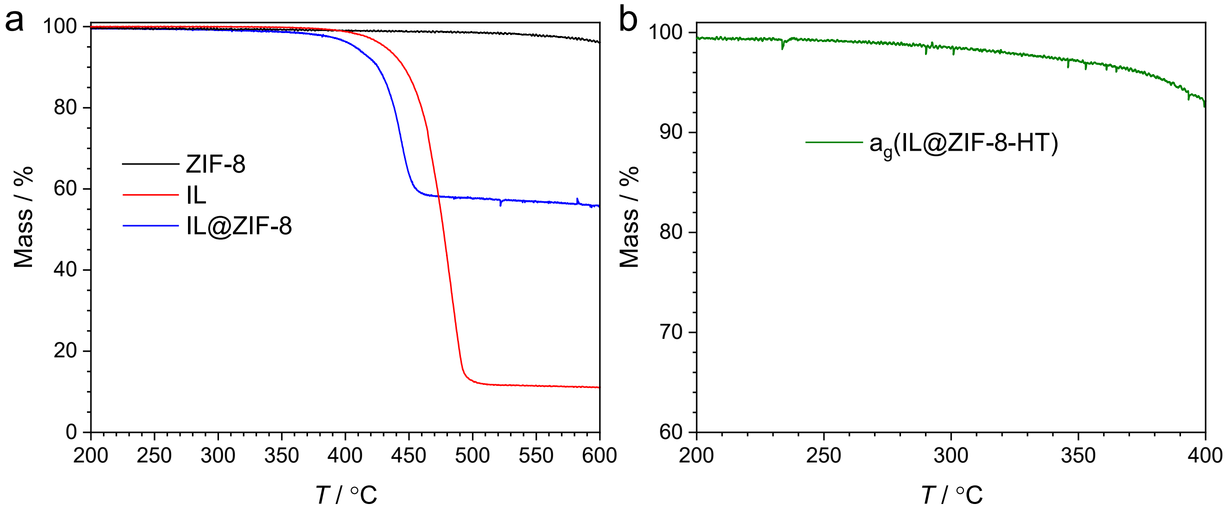

Supplementary Figure 2. Thermogravimetric analysis. (**a**) ZIF-8, IL, and IL@ZIF-8 composite. (**b**) a_g(IL@ZIF-8)-HT. A heating rate of 5 °C·min^{-1} was used to obtain the TG-DSC scans. Corresponding DSC scans are shown in Figure 2c.

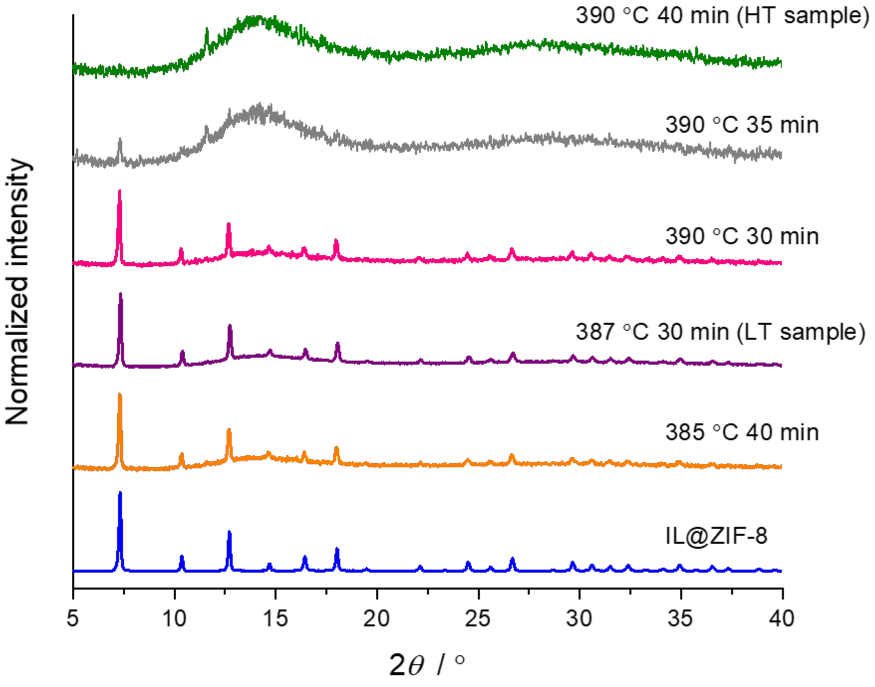

Supplementary Figure 3. X-ray diffraction patterns on IL@ZIF-8 composites following various heat-treatment conditions. Conditions for the LT and HT samples were selected from this initial screening procedure (as labelled).

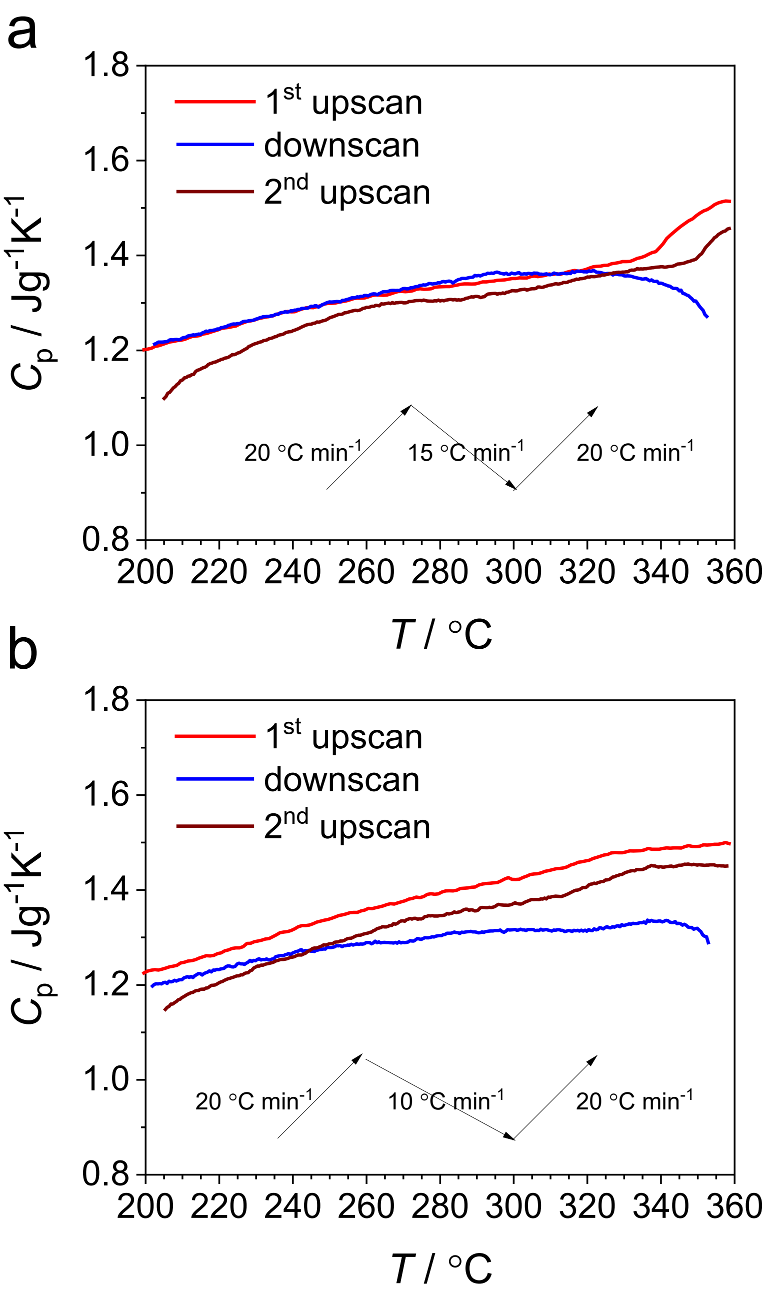

Supplementary Figure 4. Cyclic DSC (C_p) scans of a_g(IL@ZIF-8-HT) using different cooling rates between two consecutive heating scans. (**a**) Cooling rate of 15 °C ·min^{-1}. (**b**) Cooling rate of 10 °C·min^{-1}. Insets show the respective heating and cooling rates.

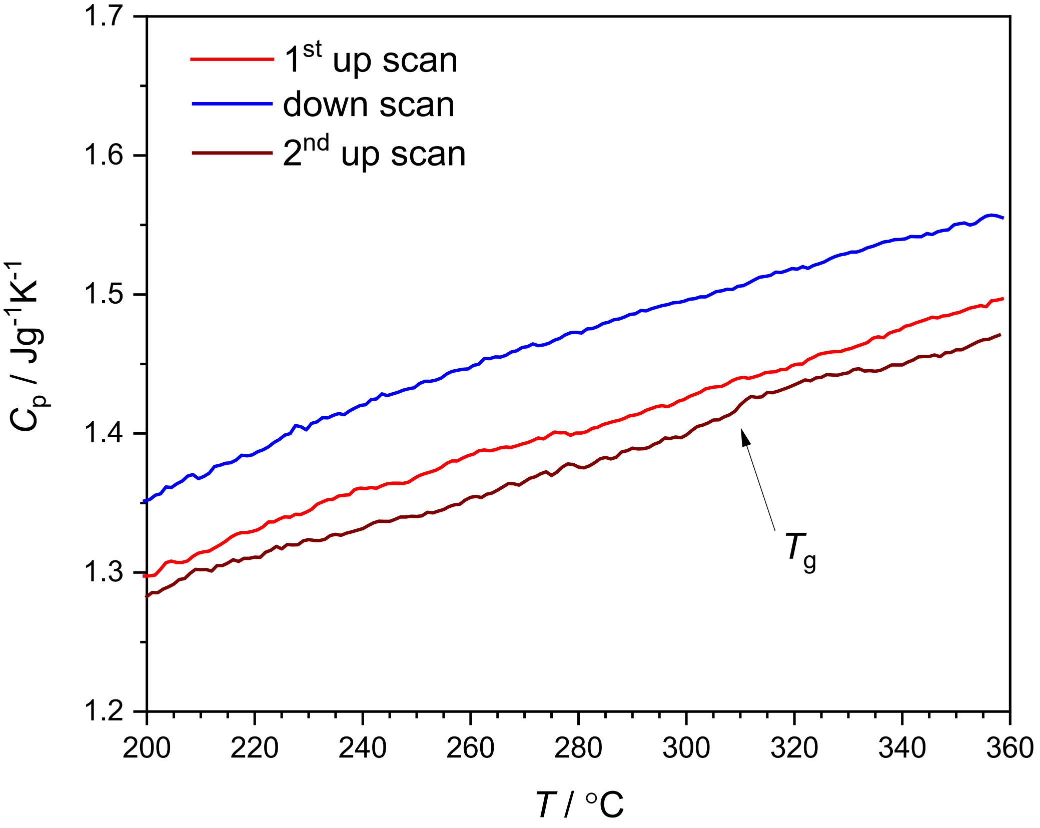

Supplementary Figure 5. Cyclic DSC (C_p) scan of a_g(IL@ZIF-8-LT) obtained by heating-cooling-heating with a same rate of 20 °C·min^{-1}.

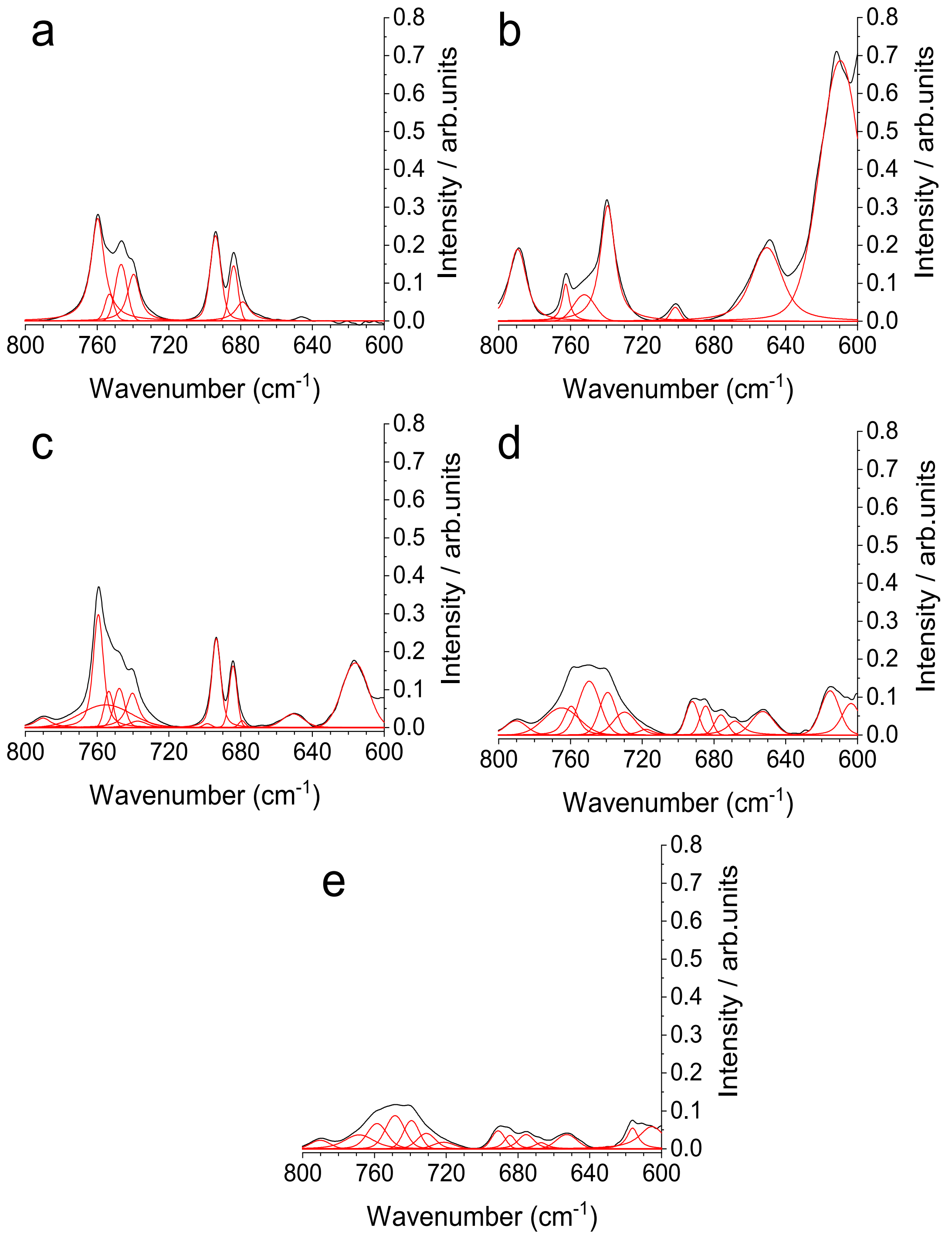

Supplementary Figure 6. Deconvoluted IR spectra in the region 600-800 cm^{-1}, out of plane bending of imidazole ring. (**a**) ZIF-8. (**b**) IL. (**c**) IL@ZIF-8. (**d**) a_g(IL@ZIF-8-LT). (**e**) a_g(IL@ZIF-8-HT). Peaks are deconvoluted in Fityk using a Voigt function.[10]

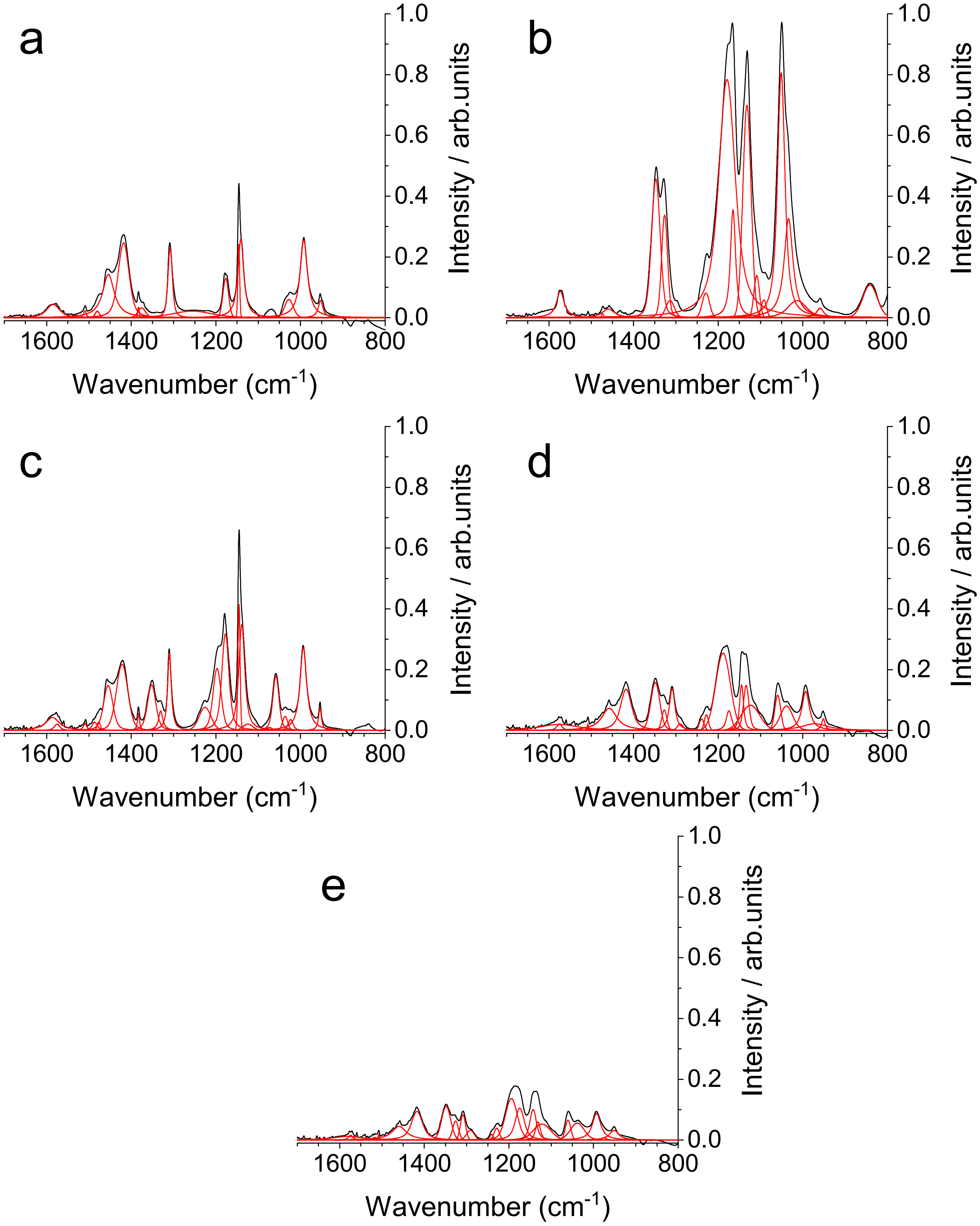

Supplementary Figure 7. Deconvoluted IR spectra in the region 800-1700 cm^{-1}, in-plane bending and entire ring stretching of imidazole ring. (**a**) ZIF-8. (**b**) IL. (**c**) IL@ZIF-8. (**d**) a_g(IL@ZIF-8-LT). (**e**) a_g(IL@ZIF-8-HT). Peaks are deconvoluted in Fityk using a Voigt function.[10]

Supplementary Table 1. IR shifts in IL@ZIF-8, a_g(IL@ZIF-8-LT), and a_g(IL@ZIF-8-HT) compared to pristine ZIF-8 in the region 600-800 cm^{-1} (out of plane bending of imidazole ring). Shifts are calculated by subtracting peak positions in crystalline and glassy composite from the corresponding peaks in ZIF-8.

ZIF-8	600-800 cm^{-1} shift in IR peaks compared to ZIF-8		
	IL@ZIF-8	ag(IL@ZIF-8-LT)	ag(IL@ZIF-8-HT)
679.04	0.17	-10.78	-11.97
683.94	0.3	-7.83	-8.46
694.02	-0.42	-9.38	-9.53
739.63	0.69	-9.8	-8.53
746.78	0.87	-7.53	-7.41
753.06	0.45	-3.52	-4.55
759.54	-0.19	0.02	-0.96

Supplementary Table 2. IR shifts in IL@ZIF-8, a_g(IL@ZIF-8-LT), and a_g(IL@ZIF-8-HT) compared to pristine ZIF-8 in the region 800-1700 cm^{-1} (in-plane bending and entire ring stretching of imidazole ring). Shifts are calculated by subtracting peak positions in crystalline and glassy composite from the corresponding peaks in ZIF-8.

ZIF-8	800-1700 cm^{-1} shift in IR peaks compared to ZIF-8		
	IL@ZIF-8	ag(IL@ZIF-8-LT)	ag(IL@ZIF-8-HT)
952.59	1.46	-0.94	-1.13
992.36	1.06	0.95	0.52
1145.97	-0.58	-1.12	-3.17
1176.76	0.81	-2.25	-2.41
1308.06	1.7	0.74	0.55
1418.13	3.53	-0.44	-0.84
1453.97	0.63	3.56	5.57
1480.28	-3.49	4.24	5.17
1509.6	-0.28	-1.64	-1.71
1584.79	1.48	-9.58	-10.87

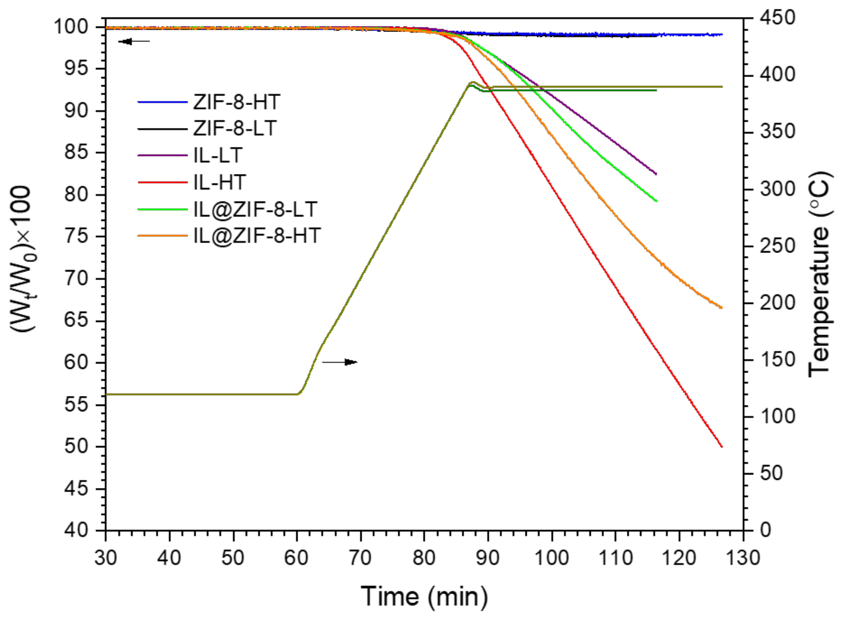

Supplementary Figure 8. Thermal stability of bulk IL, pristine ZIF-8, and IL@ZIF-8 composite at melting conditions of 387 °C 30 minutes and 390 °C 40 minutes corresponding to LT and HT, respectively. Olive and dark green lines related to y-axis show the heat profiles used to form a_g(IL@ZIF-8-LT) and a_g(IL@ZIF-8-HT) glasses.

Supplementary Table 3. Mass losses from DSC-TGA recorded after heating bulk IL, pristine ZIF-8, and IL@ZIF-8 composite to the same melting temperatures and time of IL@ZIF-8: 387 °C 30 minutes, LT, and 390 °C 40 minutes, HT.

**Sample**	**Mass loss (%)**
IL-LT	17.5
IL-HT	50.0
ZIF-8-LT	1.0
ZIF-8-HT	0.9
IL@ZIF-8-LT	20.7
IL@ZIF-8-HT	34.4

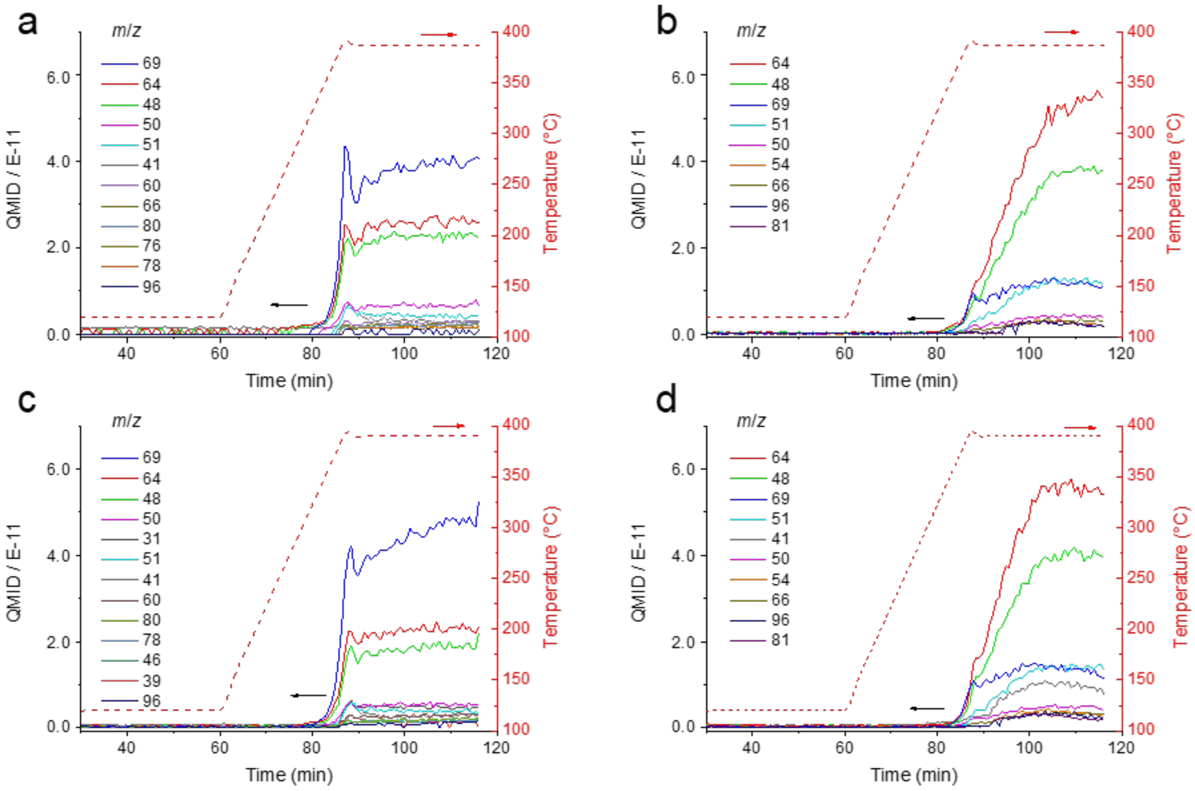

Supplementary Figure 9. TG-MS curves for LT and HT heat treatments. (**a**) IL heated at LT. (**b**) IL@ZIF-8 heated at LT. (**c**) IL heated at HT. (**d**) IL@ZIF-8 heated at HT. TG-MS experiments were performed under constant nitrogen atmosphere.

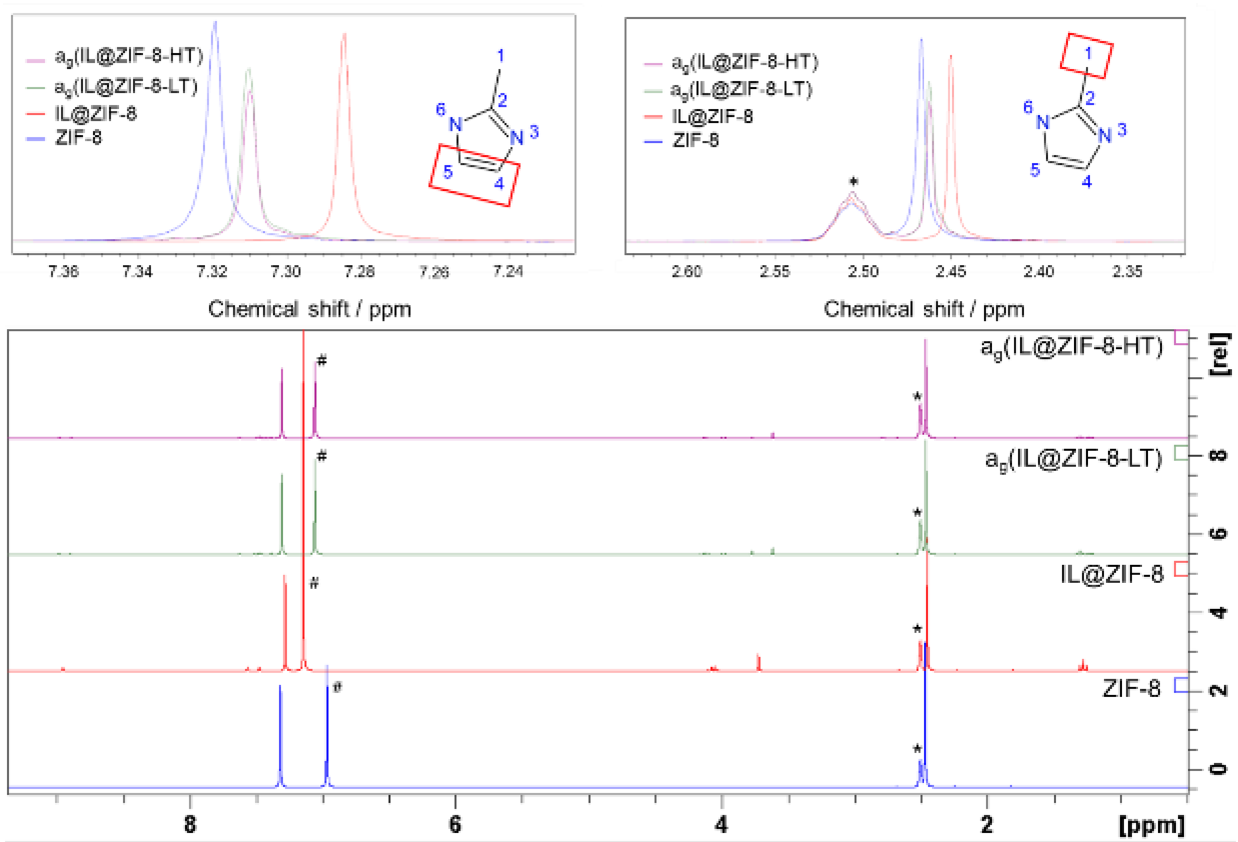

Supplementary Figure 10. ^{1}H NMR of ZIF-8, IL@ZIF-8, a$_g$(IL@ZIF-8-LT), and a$_g$(IL@ZIF-8-HT). Top figures show methyl-protons (H_1) and imidazole-protons ($H_{4,5}$) in ZIF-8. Solvent peaks are marked with asterisk. $(H,D)_3O^+$ peaks are marked with #, please see Longley *et al.*[5] for more details about solvent interactions.

Supplementary Table 4. Digested ^{1}H NMR chemical shifts of ZIF-8, IL@ZIF-8, a_g(IL@ZIF-8-LT), and a_g(IL@ZIF-8-HT) samples.

Chemical shifts / ppm				
**sample**	**H_1**	**$H_{4,5}$**	**DMSO-d_6**	**Impurity**
**ZIF-8**	2.46	7.31	2.51	6.95
**IL@ZIF-8**	2.44	7.28	2.50	7.13
**a_g(IL@ZIF-8-LT)**	2.46	7.30	2.51	7.04
**a_g(IL@ZIF-8-HT)**	2.46	7.29	2.51	7.05

Supplementary Table 5. $H_{4,5}/H_1$ ratio from ^{1}H NMR integration of imidazole and methyl protons.

Intensity relative to DMSO-d_6			
**sample**	**H_1**	**$H_{4,5}$**	**$H_{4,5}/H_1$**
**ZIF-8**	1.91	1.29	0.67
**IL@ZIF-8**	1.26	0.84	0.66
**a_g(IL@ZIF-8-LT)**	1.28	0.76	0.59
**a_g(IL@ZIF-8-HT)**	1.11	0.62	0.55

Supplementary Table 6. Intensity of H_1 and $H_{4,5}$ singlets in IL@ZIF-8, a_g(IL@ZIF-8-LT), and a_g(IL@ZIF-8-HT) with respect to pristine ZIF-8.

**Intensity relative to pure ZIF-8**		
**sample**	**H_1**	**$H_{4,5}$**
**ZIF-8**	100.00	100.00
**IL@ZIF-8**	65.95	65.29
**a_g(IL@ZIF-8-LT)**	66.79	59.09
**a_g(IL@ZIF-8-HT)**	58.15	48.10

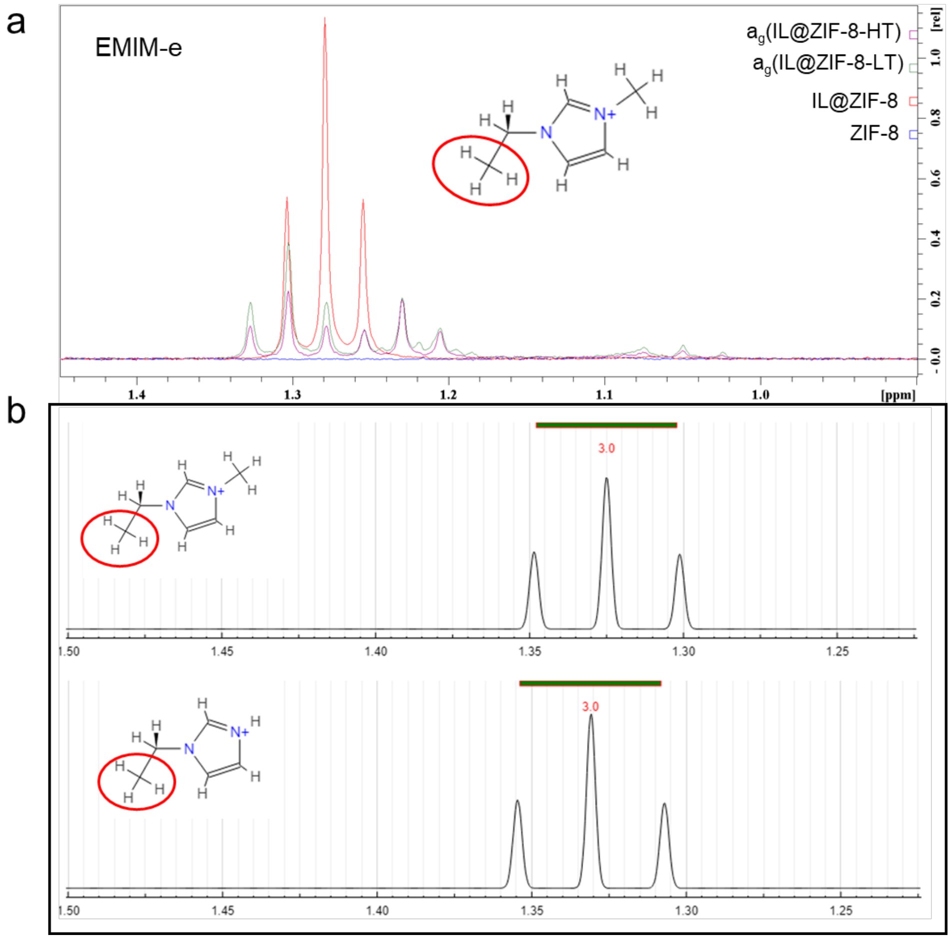

Supplementary Figure 11. ^{1}H NMR of EMIM protons. (**a**) ^{1}H NMR of EMIM-e protons. (**b**) Predicted ^{1}H NMR of EMIM-e with possible decomposed structure. Prediction of ^{1}H NMR spectra was done using *www.nmrdb.org*.[11,12]

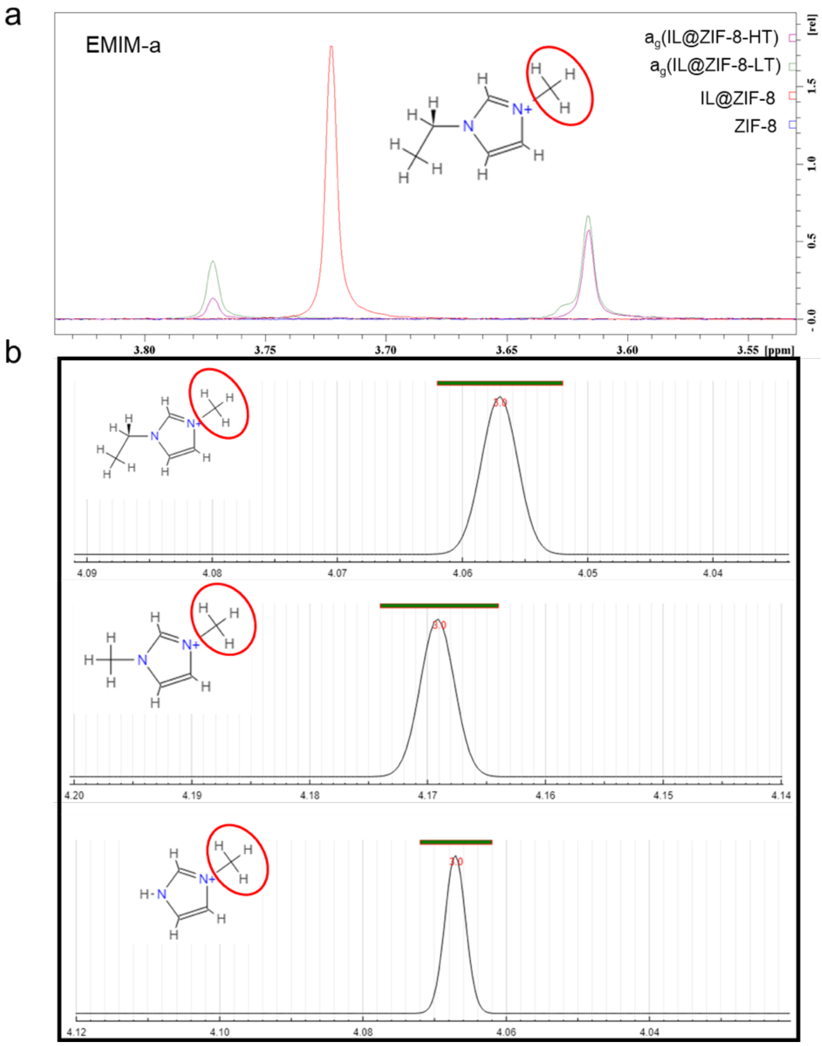

Supplementary Figure 12. ^{1}H NMR of EMIM protons. (**a**) ^{1}H NMR of EMIM-a protons. (**b**) Predicted ^{1}H NMR of EMIM-a with possible decomposed structure. Prediction of ^{1}H NMR spectra was done using *www.nmrdb.org*.[11,12]

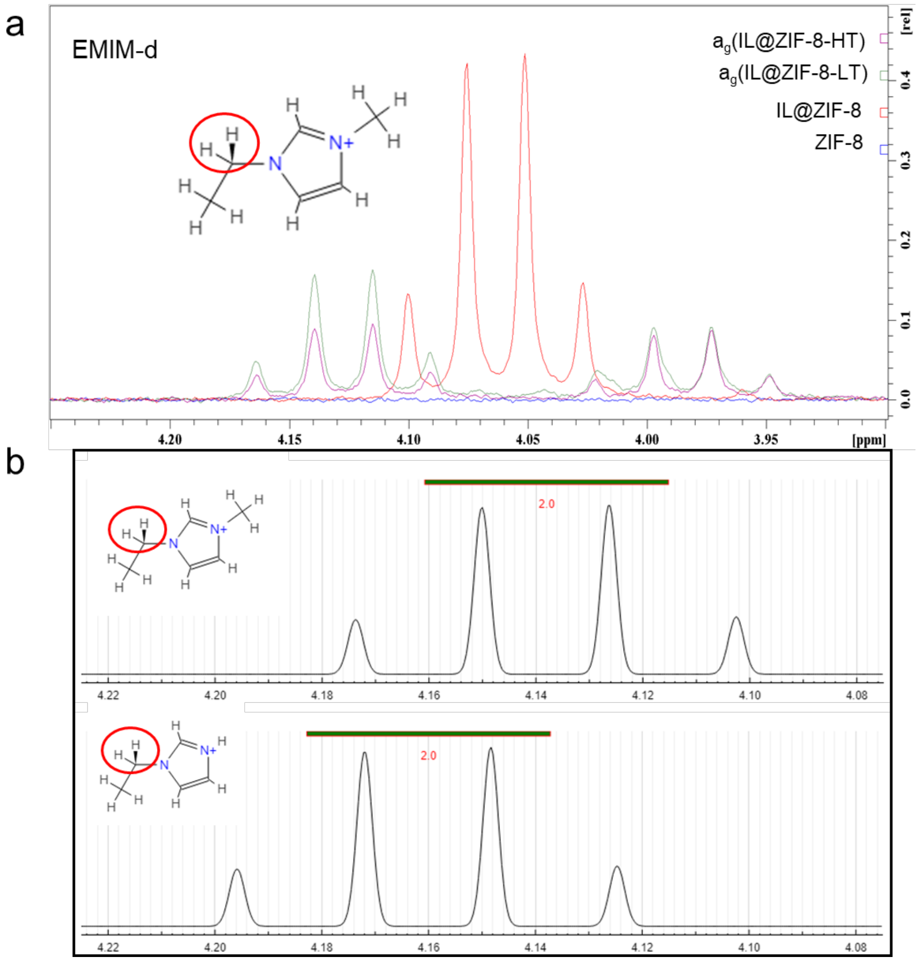

Supplementary Figure 13. ^{1}H NMR of EMIM protons. (**a**) ^{1}H NMR of EMIM-d protons. (**b**) Predicted ^{1}H NMR of EMIM-d with possible decomposed structure. Prediction of ^{1}H NMR spectra was done using *www.nmrdb.org*.[11,12]

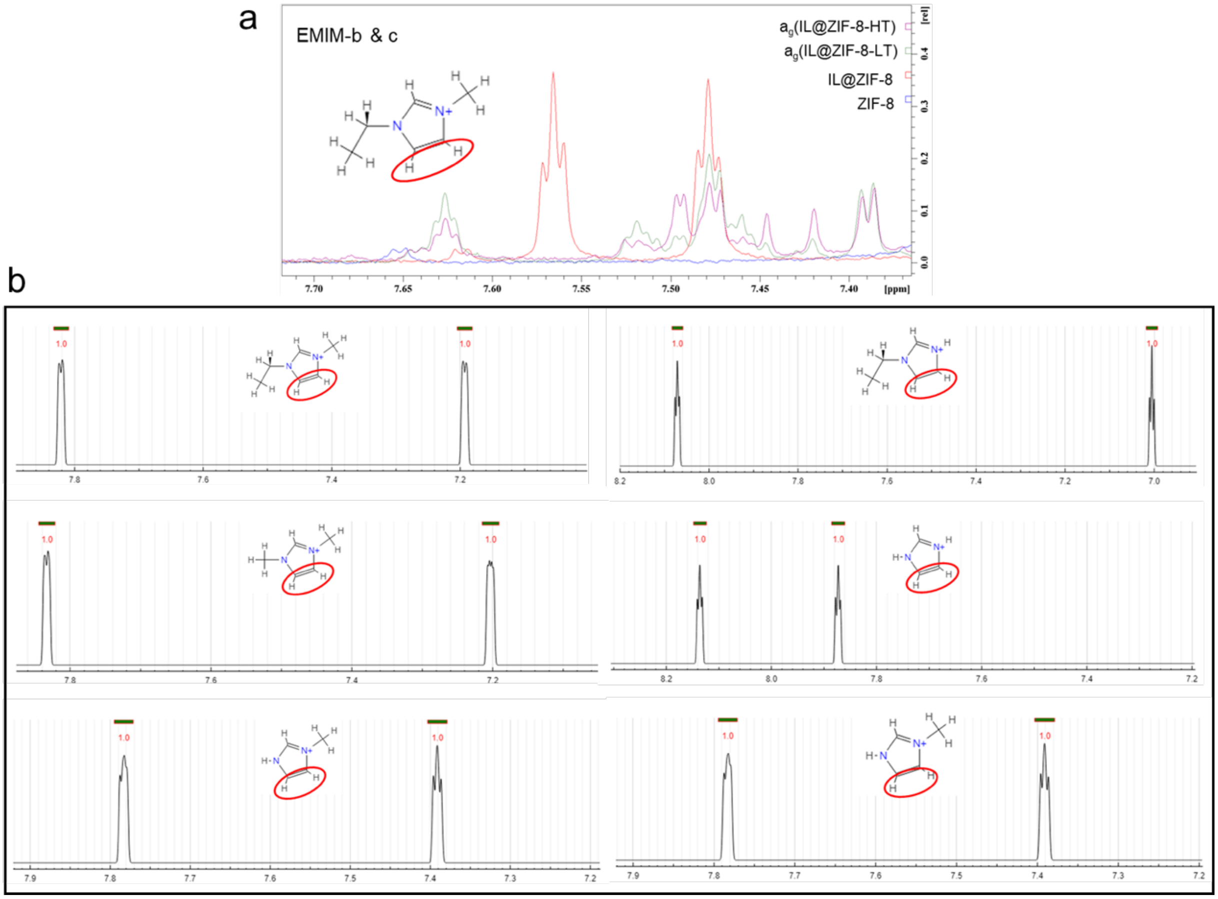

Supplementary Figure 14. ^{1}H NMR of EMIM protons. (**a**) ^{1}H NMR of EMIM-b & c protons. (**b**) Predicted ^{1}H NMR of EMIM-b & c with possible decomposed structure. Prediction of ^{1}H NMR spectra was done using *www.nmrdb.org*.[11,12]

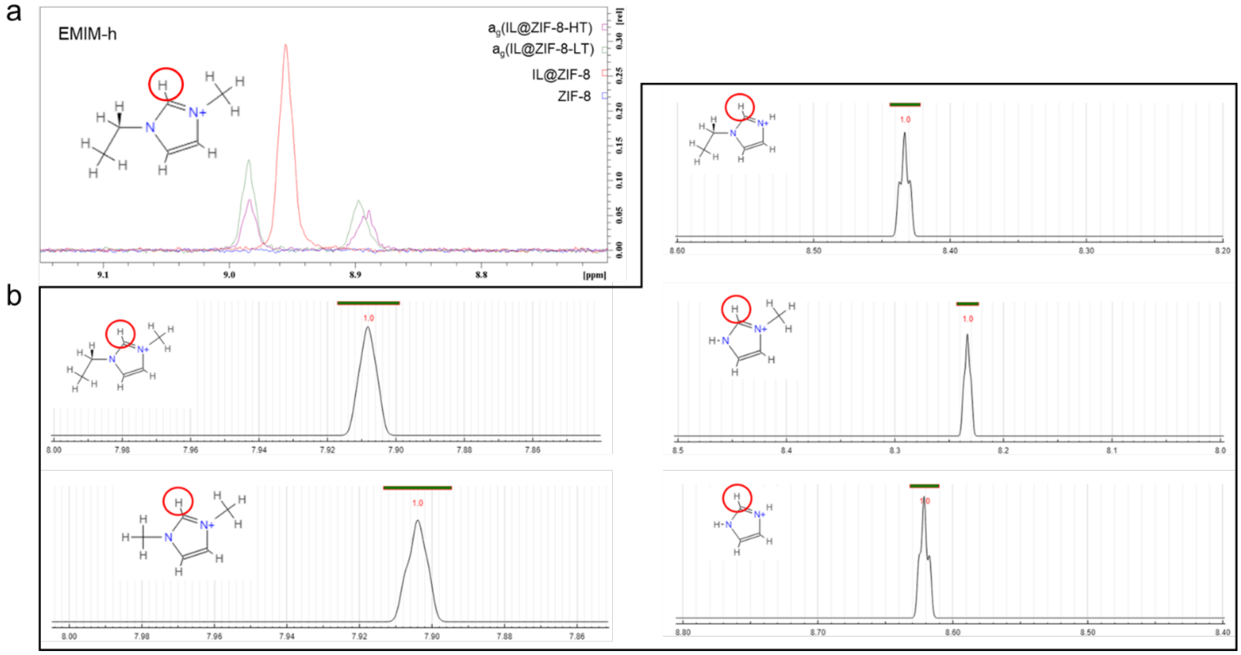

Supplementary Figure 15. ^{1}H NMR of EMIM protons. (**a**) ^{1}H NMR of EMIM-h protons. (**b**) Predicted ^{1}H NMR of EMIM-h with possible decomposed structure. Prediction of ^{1}H NMR spectra was done using *www.nmrdb.org*.[11,12]

Supplementary Table 7. Digested ^{1}H NMR chemical shifts of IL protons in IL@ZIF-8, a_g(IL@ZIF-8-LT), and a_g(IL@ZIF-8-HT) samples.

Chemical shifts / ppm						
**sample**	**EMIM-h**	**EMIM-b & c**	**EMIM-d**	**EMIM-a**	**EMIM-e**	**DMSO-d$_6$**
**IL@ZIF-8**	8.95	7.47/7.56	4.05	3.71	1.27	2.50
**a_g(IL@ZIF-8-LT)**	8.89/8.98	-	3.98/4.12	3.61/3.77	1.09/1.22/1.30	2.51
**a_g(IL@ZIF-8-HT)**	8.88/8.98	-	3.98/4.12	3.61/3.77	1.06/1.22/1.30	2.51

Supplementary Table 8. Intensity of IL protons in IL@ZIF-8, a_g(IL@ZIF-8-LT), and a_g(IL@ZIF-8-HT) with respect to EMIMTFSI protons in IL@ZIF-8.

Intensity relative to EMIMTFSI protons					
**sample**	**EMIM-h**	**EMIM-b & c**	**EMIM-d**	**EMIM-a**	**EMIM-e**
**IL@ZIF-8**	100.00	100.00	100.00	100.00	100.00
**a_g(IL@ZIF-8-LT)**	56.47	97.55	64.89	62.63	77.96
**a_g(IL@ZIF-8-HT)**	38.64	97.20	41.49	39.50	49.72

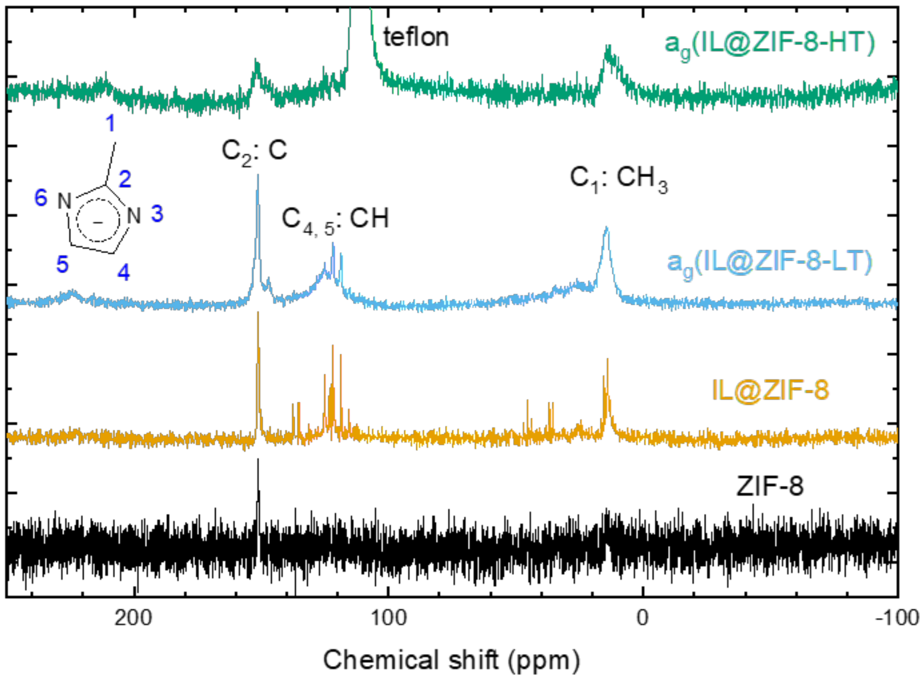

Supplementary Figure 16. ^{13}C single pulse of ZIF-8, IL@ZIF-8, a_g (IL@ZIF-8-LT) and a_g(IL@ZIF-8-HT).

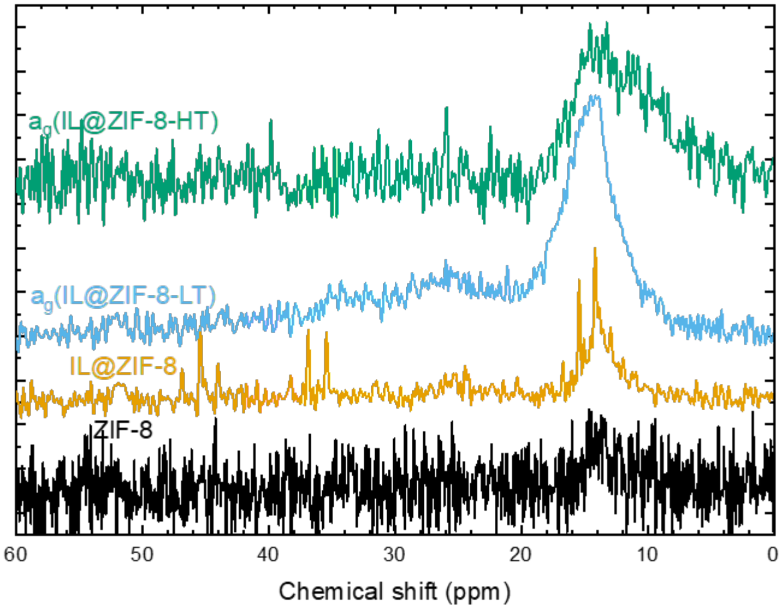

Supplementary Figure 17. ^{13}C of C_1 or CH_3 in ZIF-8, IL@ZIF-8, a_g (IL@ZIF-8-LT) and a_g(IL@ZIF-8-HT).

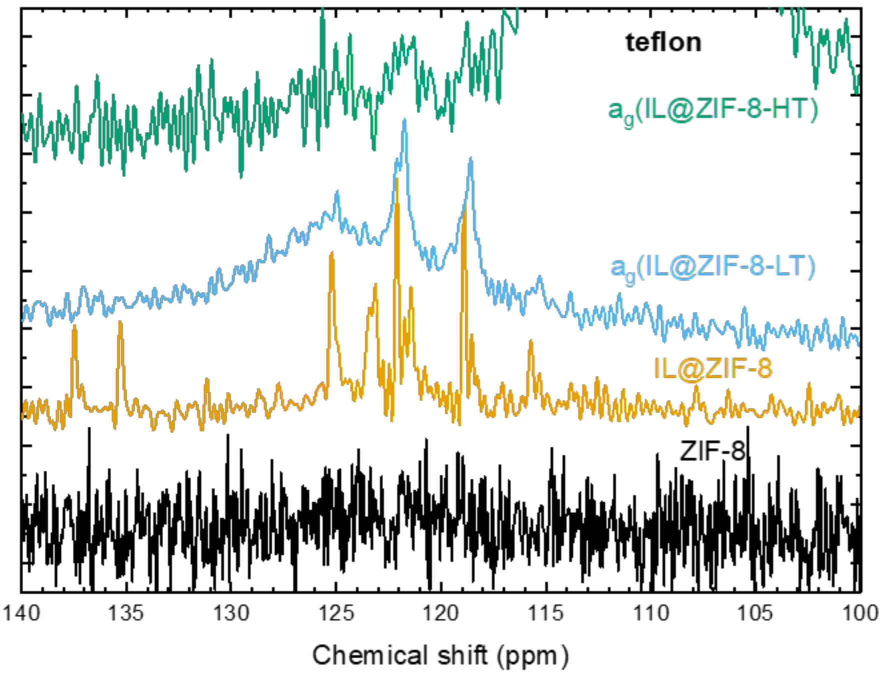

Supplementary Figure 18. [13]C of $C_{4,\ 5}$ or CH in ZIF-8, IL@ZIF-8, a_g (IL@ZIF-8-LT) and a_g(IL@ZIF-8-HT).

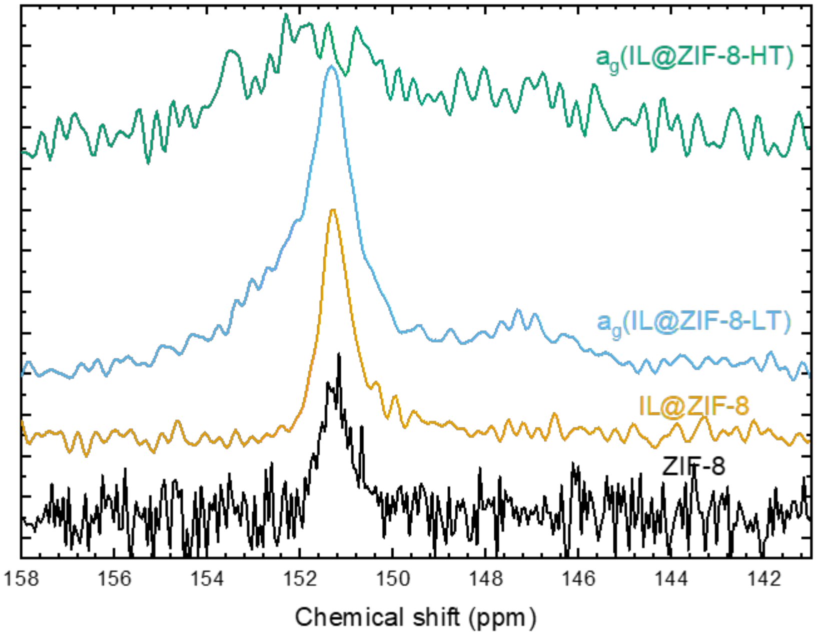

Supplementary Figure 19. [13]C of C_2 or lone C in ZIF-8, IL@ZIF-8, a_g (IL@ZIF-8-LT) and a_g(IL@ZIF-8-HT).

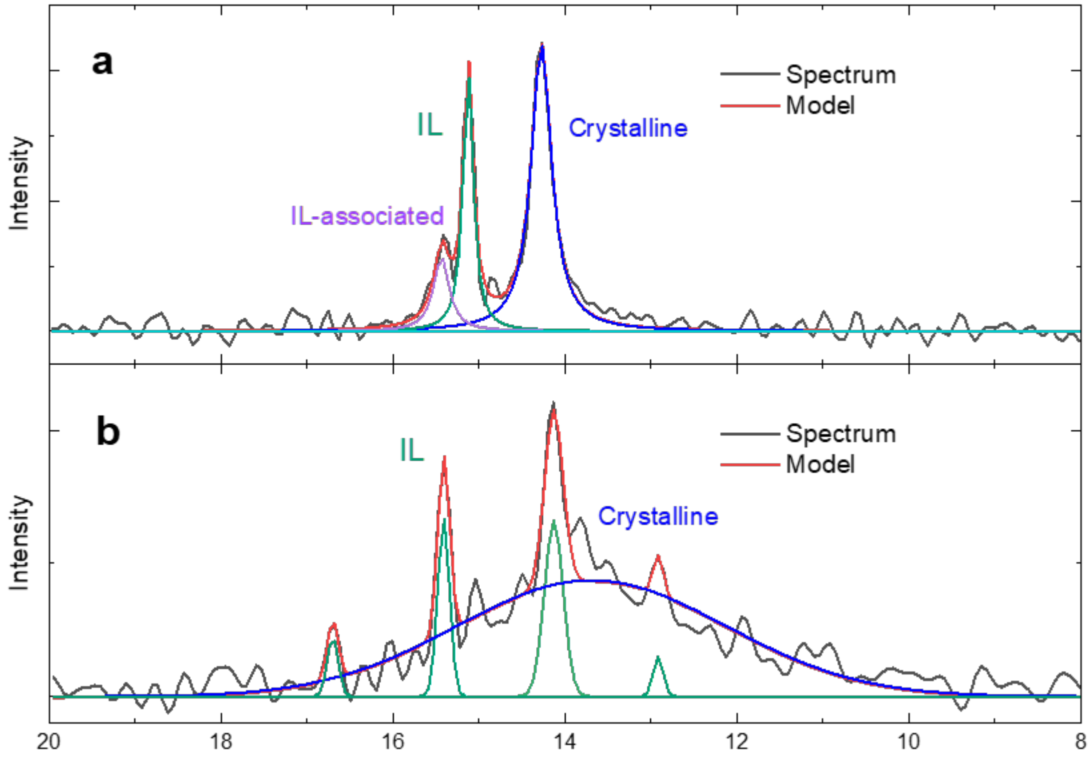

Supplementary Figure 20. Comparison of fitting of single-pulse ^{13}C NMR of the C_1 or CH_3 region in ZIF-8 (**a**) decoupled and (**b**) coupled. Both fits have the same approximate amount of IL and ZIF-8.

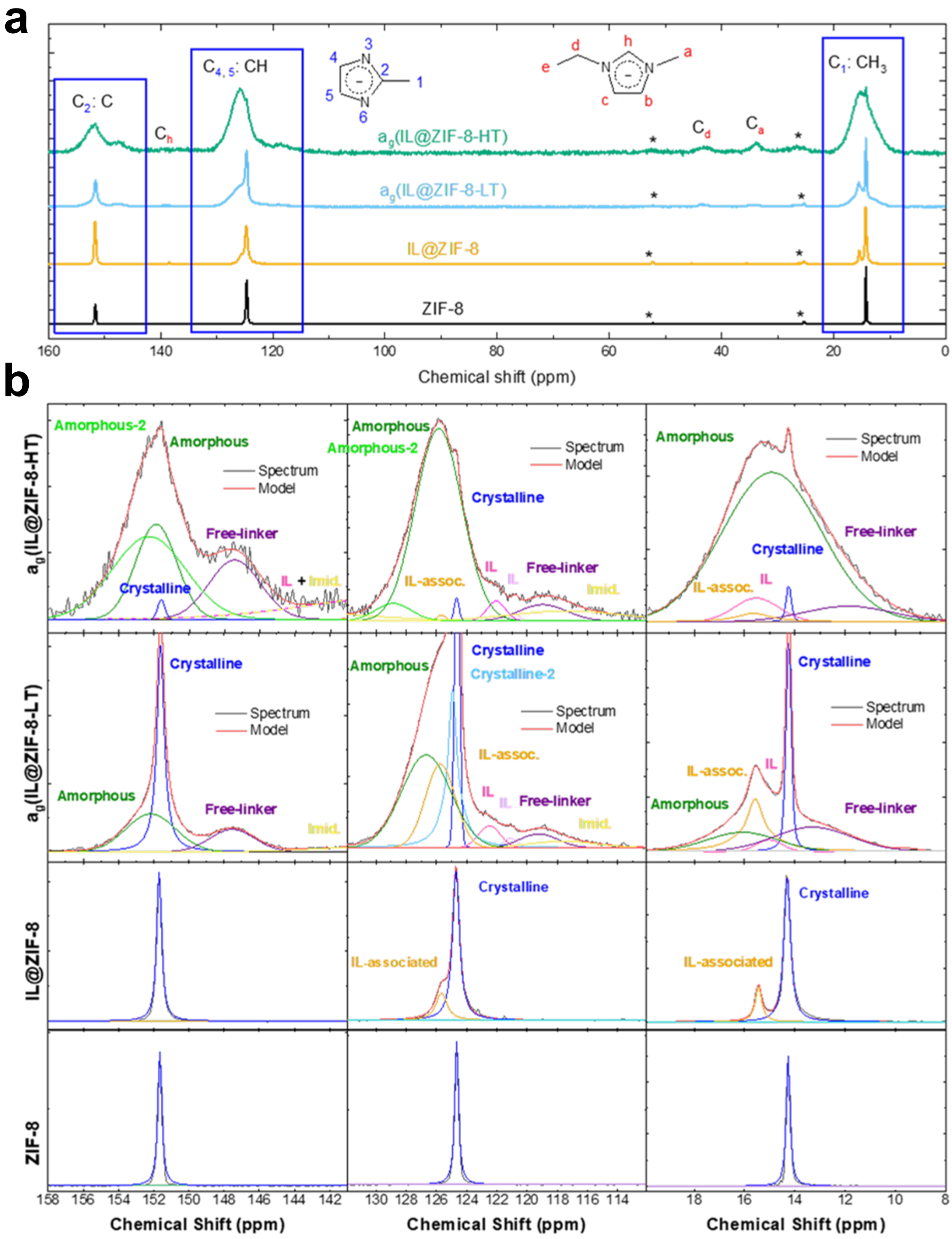

Supplementary Figure 21. **^{1}H-^{13}C CP NMR analysis of mIm carbons in ZIF-8**. (**a**) ^{1}H-^{13}C CP NMR of ZIF-8, IL@ZIF-8, a_g(IL@ZIF-8-LT) and a_g(IL@ZIF-8-HT). Spinning sidebands are marked with asterisks. (**b**). Insets of fits for C_2:C, $C_{4,5}$:CH and C_1: CH_3 (methyl) carbons.

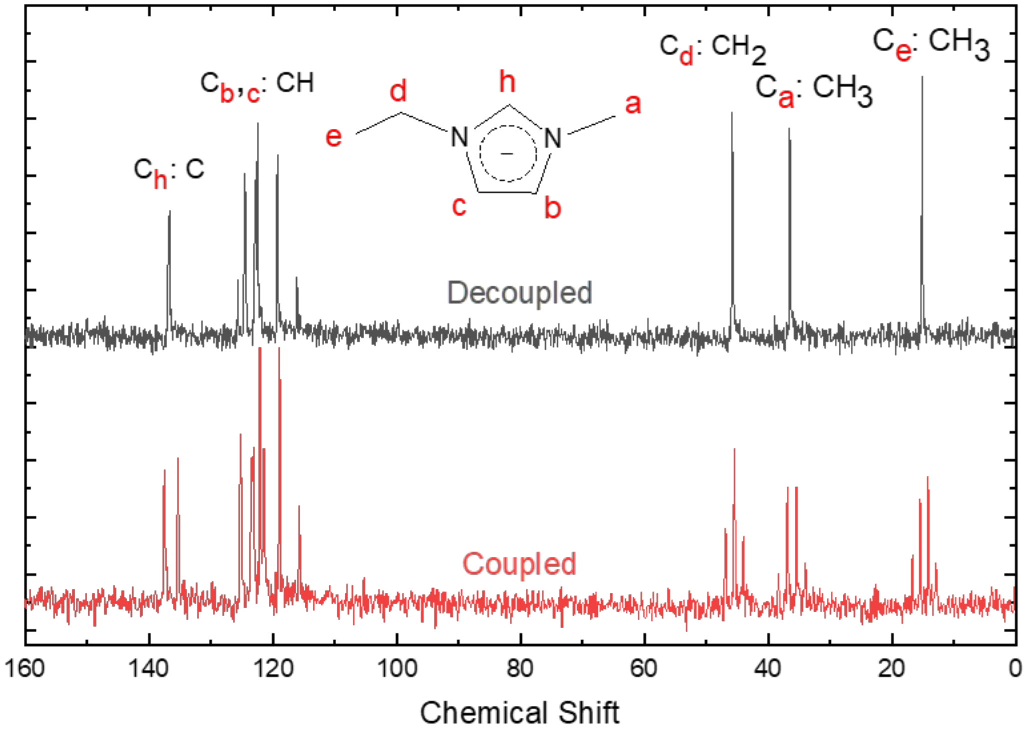

Supplementary Figure 22. ^{13}C single-pulse of IL cation, EMIM. Decoupled vs. pulse sequence with no decoupling.

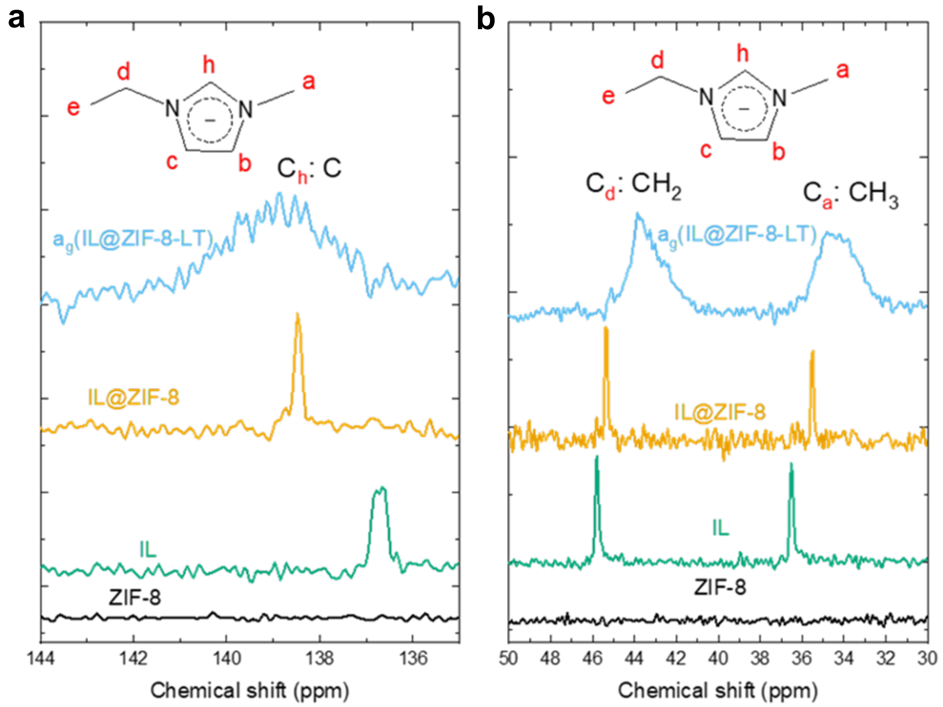

Supplementary Figure 23. ^1H–^{13}C CP NMR. (**a**) C_h or C (**b**) C_a:CH_3 and C_d:CH_2 . CH_3 regions in IL cation EMIM.

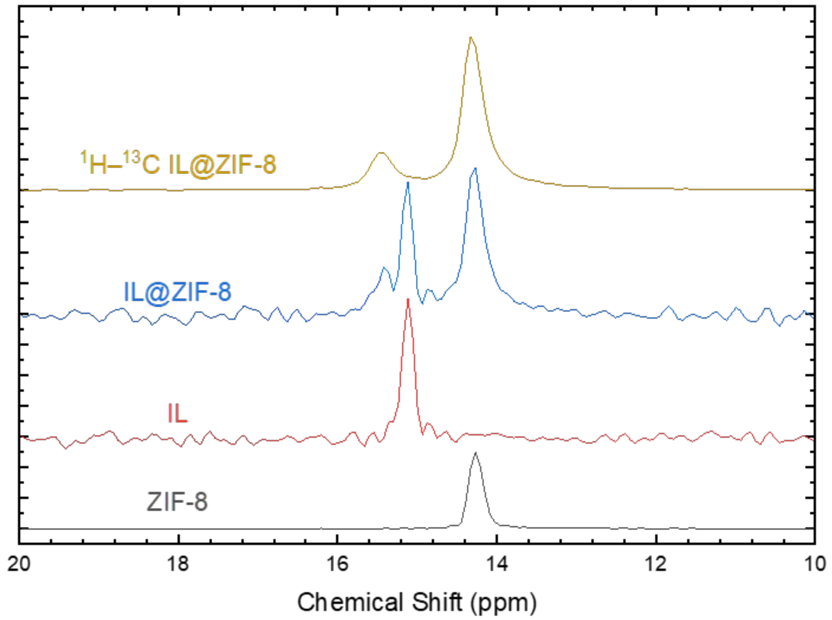

Supplementary Figure 24. ^{13}C and ^1H–^{13}C CP NMR of CH_3 regions in ZIF-8.

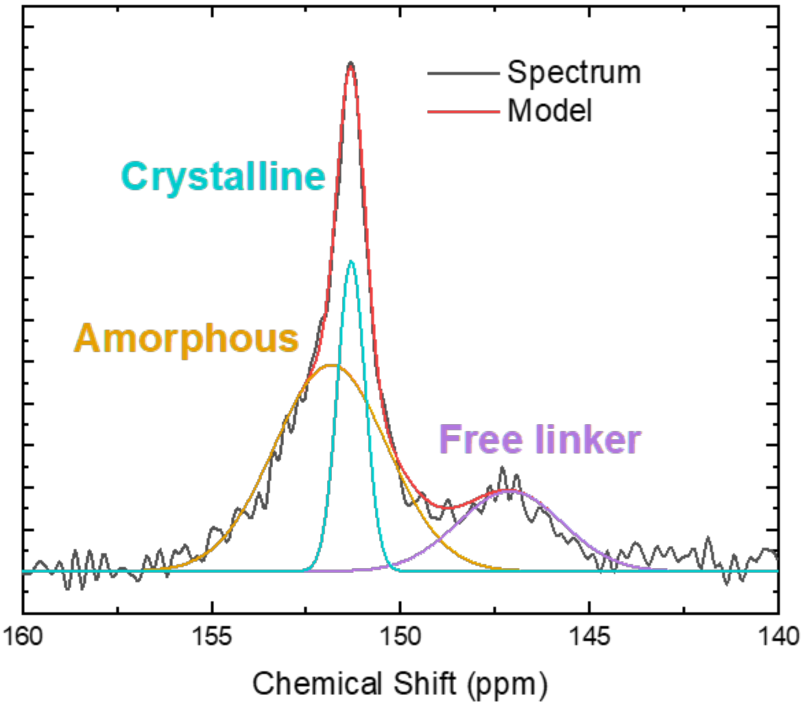

Supplementary Figure 25. ^{13}C single-pulse fitting of C_1 or lone C region in ZIF-8 in the LT sample.

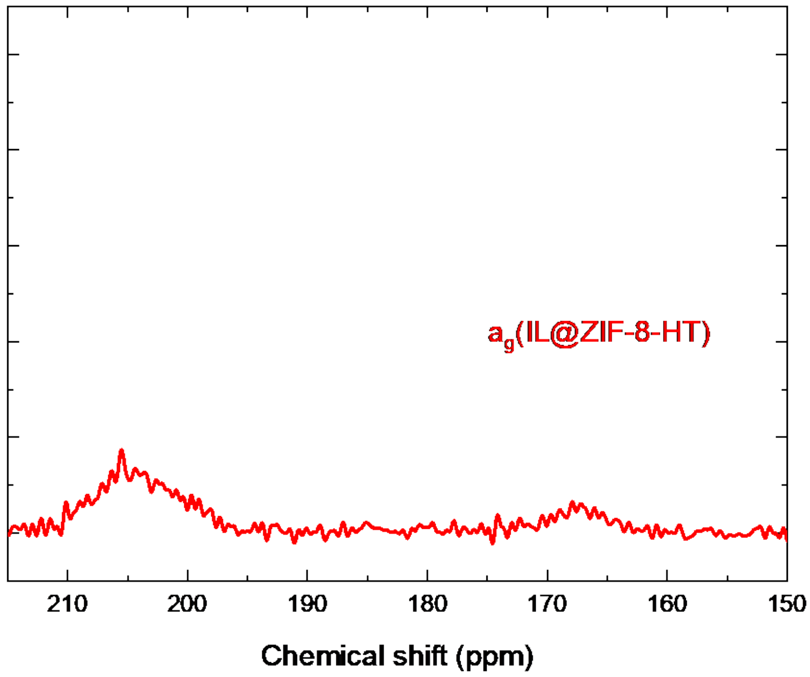

Supplementary Figure 26. ^1H-^{15}N CP NMR of a_g(IL@ZIF-8-HT).

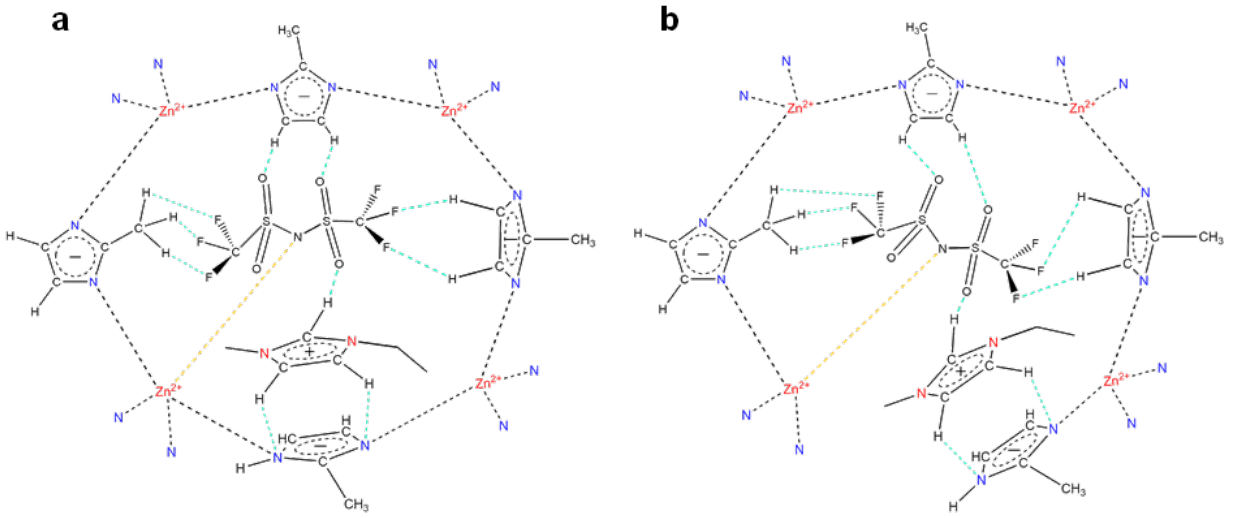

Supplementary Figure 27. Schematic of possible interactions between IL and ZIF-8. (**a**) upon IL addition (**b**) Melting/amorphization of IL@ZIF-8 at 381 °C.

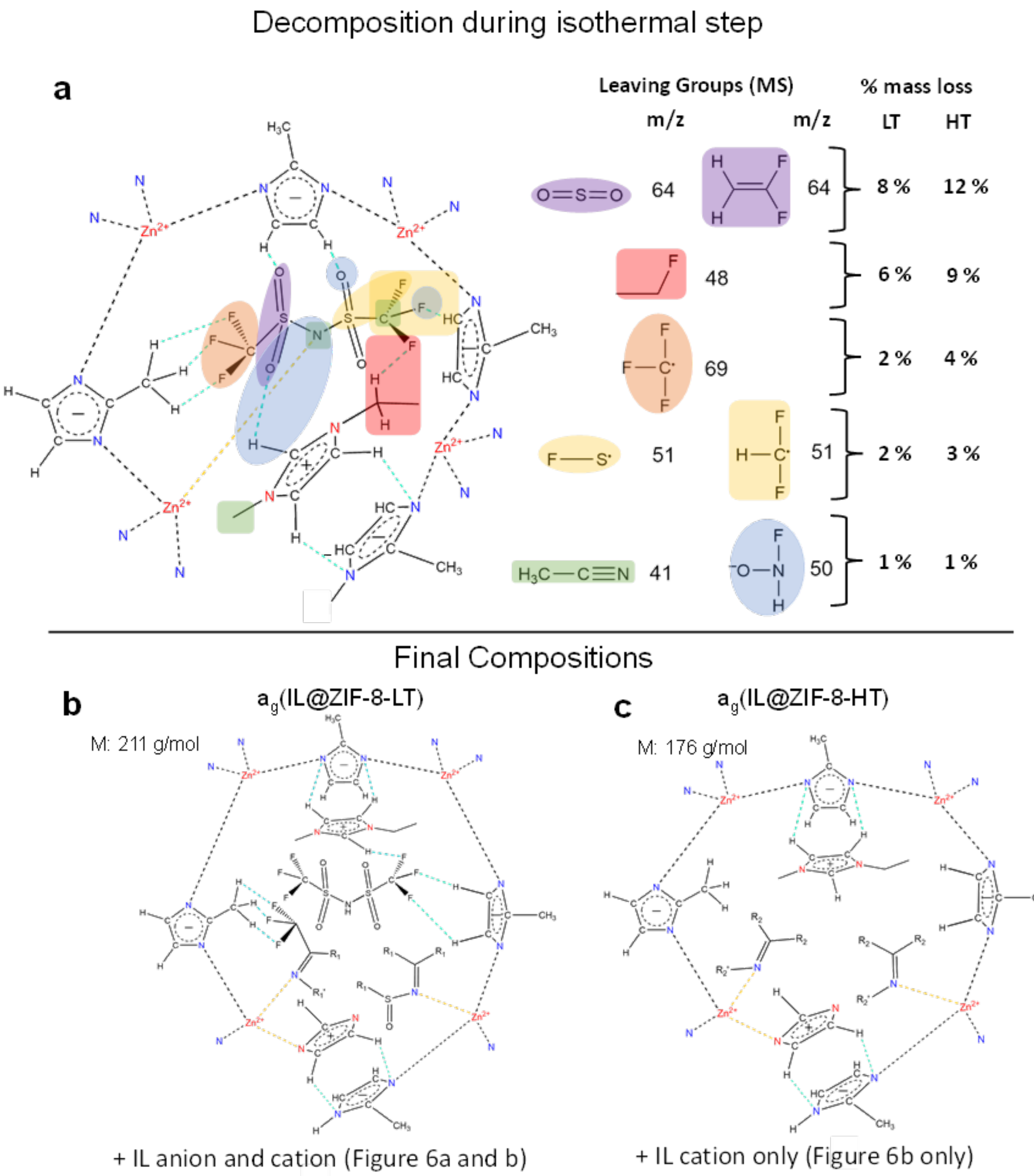

	Zn	C	N	H	F	O	S
%Ch.	+25	+8	+20	0	-48	-69	-64

	Zn	C	N	H	F	O	S
%Ch.	+54	+15	+38	-2	-99	-100	-100

Supplementary Figure 28. Route of decomposition and possible final compositions. (**a**) Likely leaving groups and their percentages of the total mass loss observed from mass spectrometry. Detected masses < 1 wt% loss are not included. Possible final compositions of (**b**) a_g(IL@ZIF-8-LT) and (**c**) a_g(IL@ZIF-8-HT) as determined from the peak area of the MS curves. R_1 is H/F or $CH_{3-x}F_x$ and R_1' is only $CH_{3-x}F_x$ and, while R_2 is only an H-containing organic group, H or CH_3 and R_2' is only CH_3.

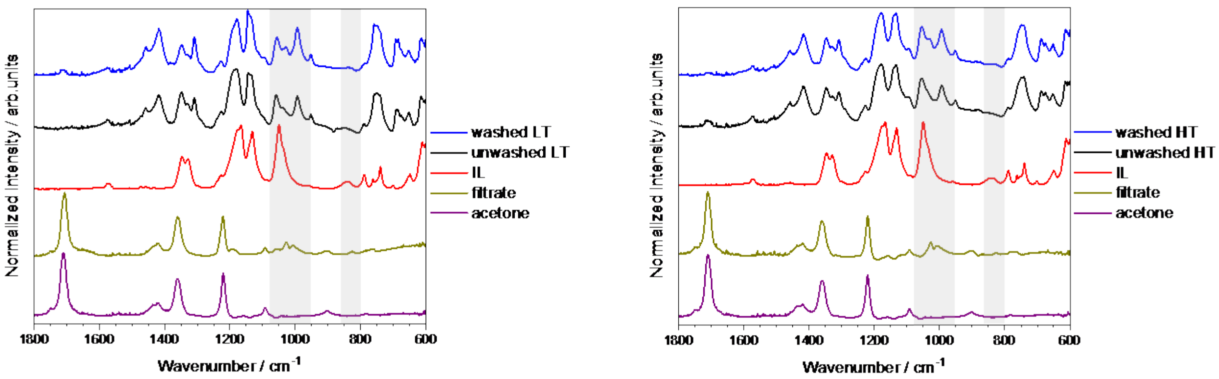

Supplementary Figure 29. FTIR spectra of a_g(IL@ZIF-8-LT) and a_g(IL@ZIF-8-HT) samples prior and after washing with acetone at 50 °C. (**a**) a_g(IL@ZIF-8-LT) sample; (**b**) a_g(IL@ZIF-8-HT) sample**.**

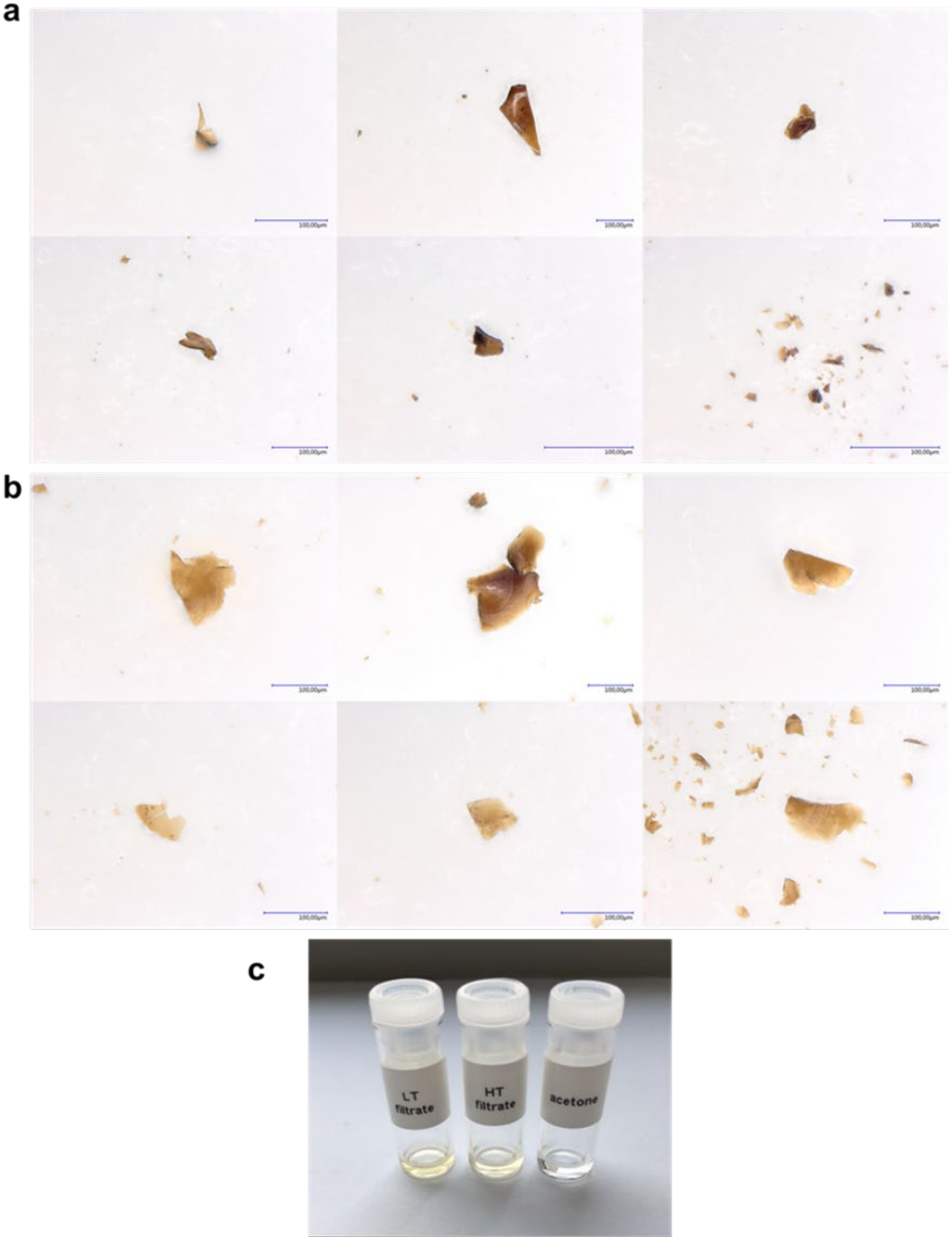

Supplementary Figure 30. Microscope images of the washed a_g(IL@ZIF-8-LT) and a_g(IL@ZIF-8-HT) samples. (**a**) washed a_g(IL@ZIF-8-HT). (**b**) washed a_g(IL@ZIF-8-LT) sample. (**c**) washing filtrates. Scale bars are 100 μm.

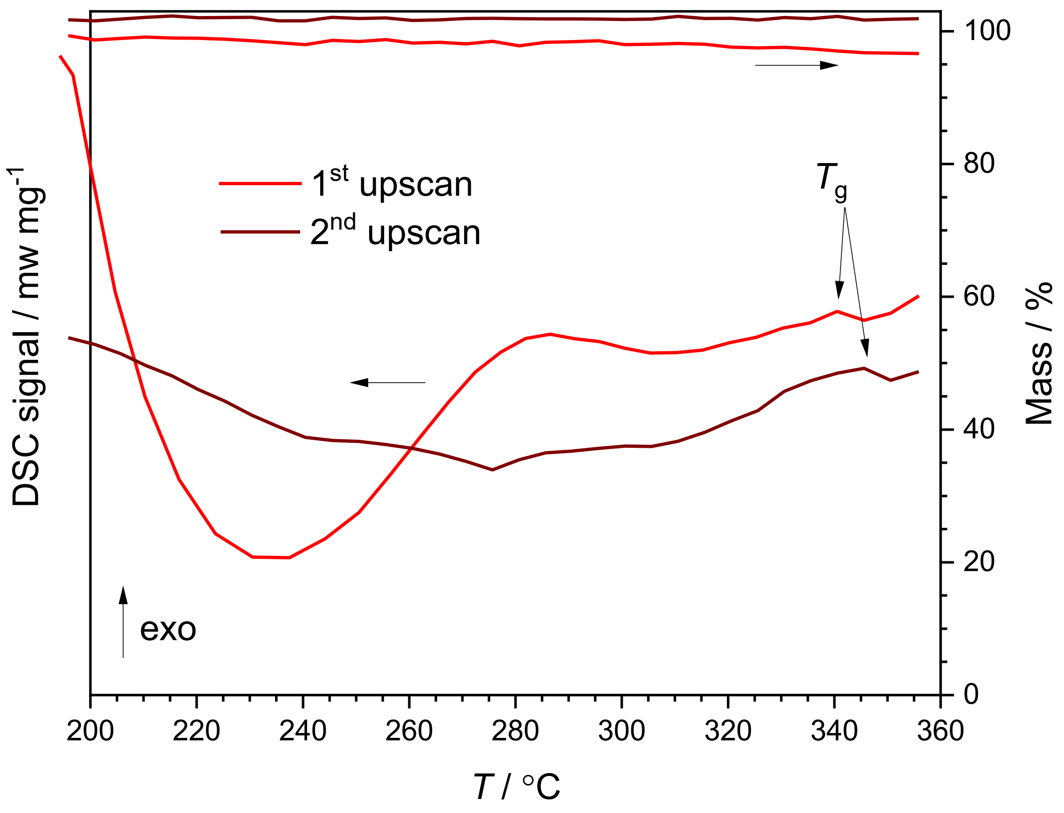

Supplementary Figure 31. DSC-TGA scan of washed a_g(IL@ZIF-8-HT) glass taken at 20 °C·min^{-1}.

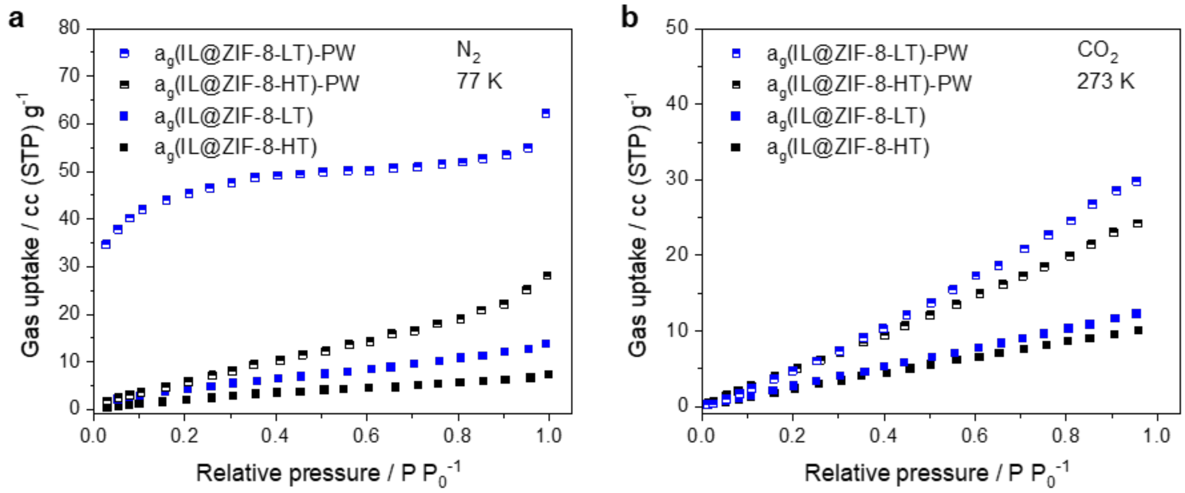

Supplementary Figure 32. Adsorption isotherms obtained for a_g(IL@ZIF-8-LT) and a_g(IL@ZIF-8-HT) samples and post-washing (IL@ZIF-8-LT)-PW and a_g(IL@ZIF-8-HT)-PW samples. (**a**) N_2 isotherms at 77 K. (**b**) CO_2 isotherms at 273 K.

Supplementary Table 9. BET Surface area and pore volume results.

sample	Surface area ($m^2\ g^{-1}$)	Pore volume ($cm^3\ g^{-1}$)
**ZIF-8**	1752	0.634
**IL@ZIF-8**	11	0.005
**a_g(IL@ZIF-8-LT)**	17	0.003
**a_g(IL@ZIF-8-HT)**	16	0.001
**a_g(IL@ZIF-8-LT)***	17	0.021
**a_g(IL@ZIF-8-HT)***	16	0.011
**a_g(IL@ZIF-8-LT)-PW***	170	0.096
**a_g(IL@ZIF-8-HT)-PW***	28	0.043
*Measured using adapted parameters. *See* Methods section for details.		

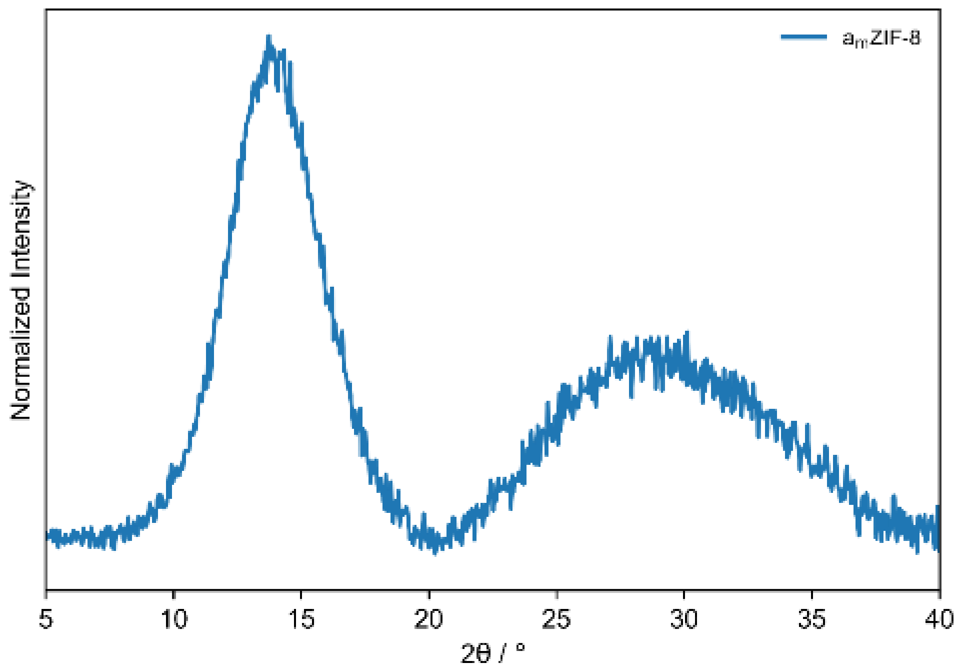

Supplementary Figure 33. Powder X-ray diffraction pattern for a sample of ZIF-8 which has been amorphized via ball-milling (30 min, 30 Hz) showing only broad features. The data have been background subtracted and normalized.

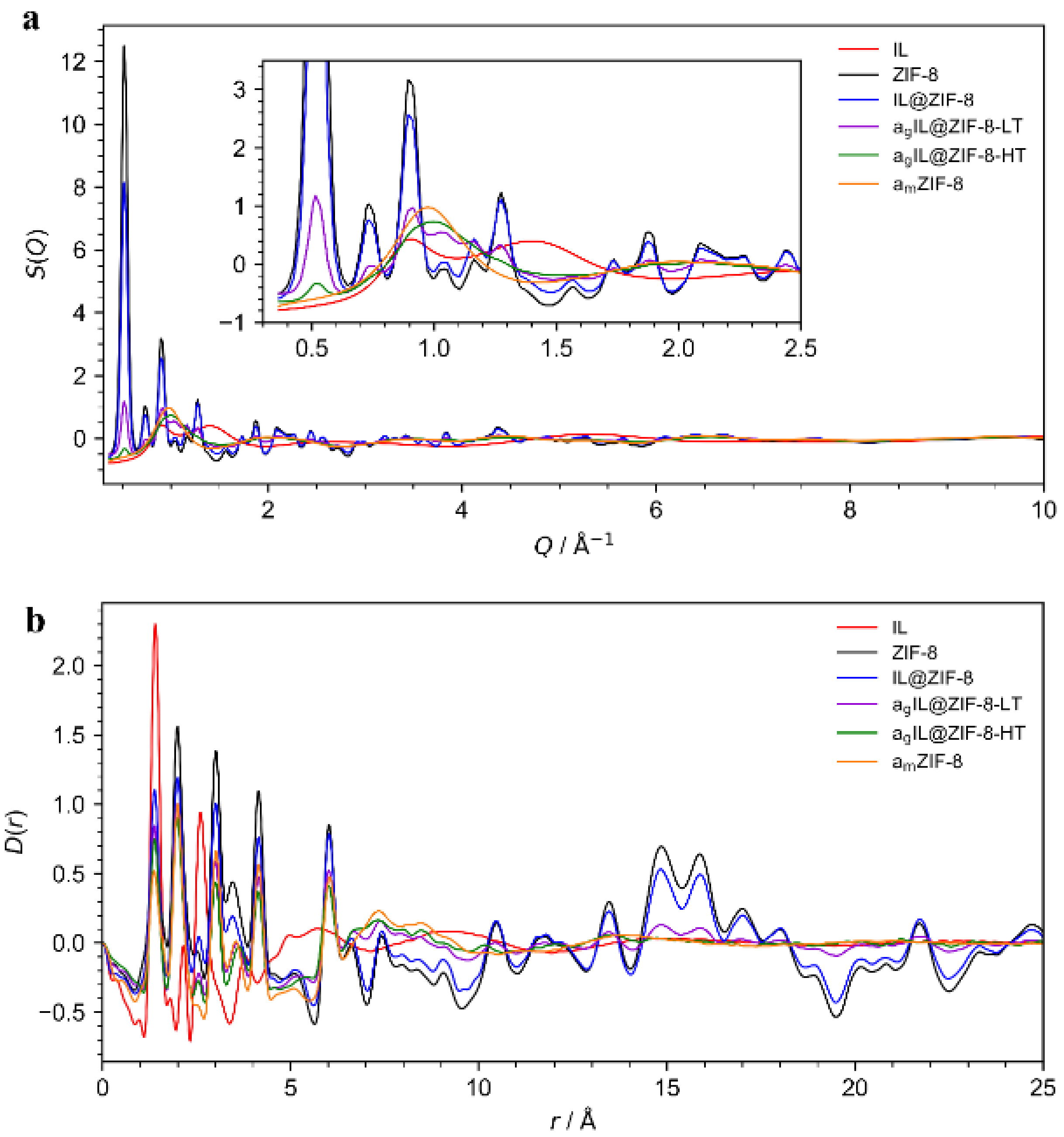

Supplementary Figure 34. Ambient temperature X-ray PDF data. (**a**) X-ray total scattering structure factor, $S(Q)$ of the IL, ZIF-8, IL@ZIF-8, a_g(IL@ZIF-8-LT), a_g(IL@ZIF-8-HT) and a_m-ZIF-8 samples. (**b**) Corresponding X-ray pair distribution functions, $D(r)$.

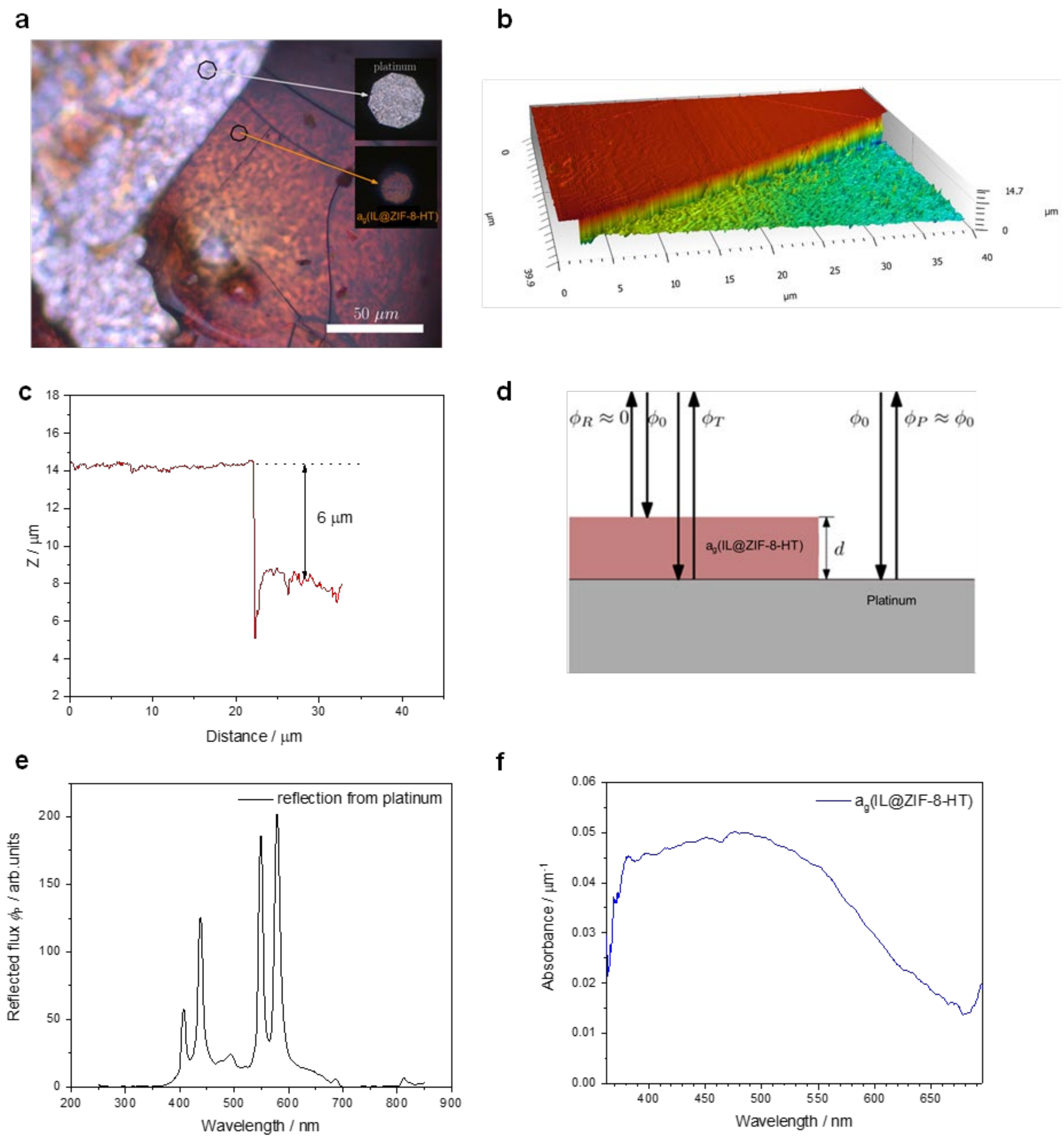

Supplementary Figure 35. Optical absorbance of a_g(IL@ZIF-8-HT) film on a platinum surface using a deuterium arc lamp. (**a-c**) Thickness determination of a_g(IL@ZIF-8-HT) using a laser scanning microscope. (**d-f**) Absorbance spectra of a_g(IL@ZIF-8-HT) film on a platinum surface.

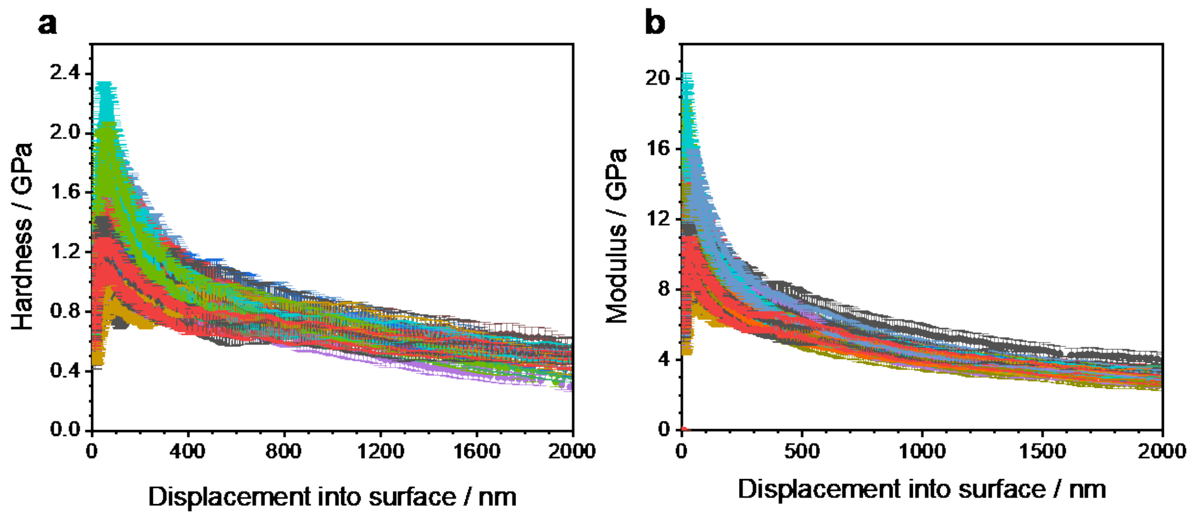

Supplementary Figure 36. Depth profiles of hardness (*H*) and modulus (*E*) for a_g(IL@ZIF-8-HT) glass obtained by instrumented indentation testing.

Supplementary References

1. Noack, K., Schulz, P. S., Paape, N. & Kiefer, J. The role of the C2 position in interionic interactions of imidazolium based ionic liquids : a vibrational and NMR spectroscopic study. *Phys. Chem. Chem. Phys* **12**, 14153–14161 (2010).

2. Wulf, A., Fumino, K. & Ludwig, R. Spectroscopic evidence for an enhanced anion-cation interaction from hydrogen bonding in pure imidazolium ionic liquids. *Angew. Chemie - Int. Ed.* **49**, 449–453 (2010).

3. Baxter, E. F. *et al.* Combined experimental and computational NMR study of crystalline and amorphous zeolitic imidazolate frameworks. *Phys. Chem. Chem. Phys.* **17**, 25191–25196 (2015).

4. Chen, Y., Li, S., Xue, Z., Hao, M. & Mu, T. Quantifying the hydrogen-bonding interaction between cation and anion of pure [EMIM][Ac] and evidencing the ion pairs existence in its extremely diluted water solution: Via 13C, 1H, 15N and 2D NMR. *J. Mol. Struct.* **1079**, 120–129 (2015).

5. Longley, L. *et al.* The Reactivity of an Inorganic Glass Melt with ZIF-8. *Dalt. Trans.* **50**, 3529–3535 (2021).

6. Hasani, M., Nordstierna, L. & Martinelli, A. Molecular dynamics involving proton exchange of a protic ionic liquid–water mixture studied by NMR spectroscopy. *Phys. Chem. Chem. Phys* **21**, 22014 (2201).

7. Longley, L. *et al.* Flux melting of metal-organic frameworks. *Chem. Sci.* **10**, 3592–3601 (2019).

8. Zhou, C. *et al.* Metal-organic framework glasses with permanent accessible porosity. *Nat. Commun.* **9**, 1–9 (2018).

9. Wang, Y. *et al.* A MOF Glass Membrane for Gas Separation. *Angew. Chem. Int. Ed.* **59**, 4365–4369 (2020).

10. Wojdyr, M. Fityk: A general-purpose peak fitting program. *J. Appl. Crystallogr.* **43**, 1126–1128 (2010).

11. Aires-de-Sousa, J., Hemmer, M. C. & Gasteiger, J. Prediction of 1H NMR chemical shifts using neural networks. *Anal. Chem.* **74**, 80–90 (2002).

12. Banfi, D. & Patiny, L. www.nmrdb.org: Resurrecting and processing NMR spectra on-line. *Chimia (Aarau)*. **62**, 280–281 (2008).
