# Supplementary material for: Ionic liquid facilitated melting of the metal-organic framework ZIF-8
Source: Nat Commun. 2021 Sep 29;12:5703. doi: 10.1038/s41467-021-25970-0 (PMC8481281; doi:10.1038/s41467-021-25970-0)
Supplement: Supplementary file 3 — Description of Additional Supplementary Files [file 41467_2021_25970_MOESM3_ESM.pdf]

Description of Additional Supplementary Files

Supplementary Movie 1. In situ heating of ZIF-8 from 25 °C to 390 °C using LSM.

Supplementary Movie 2. In situ heating of IL@ZIF-8 from 25 °C to 390 °C using LSM.
